# The Lowest Excited State of Heptacenes Is Dark

**DOI:** 10.1021/acs.jpclett.5c01314

**Published:** 2025-09-03

**Authors:** Johannes Schöntag, Philipp Frech, Kathrin Zwettler, Navid Fardan, Ankit Somani, Wolfgang Leis, Markus Ströbele, Michael Seitz, Marcus Scheele, Holger F. Bettinger

**Affiliations:** † Institut für Organische Chemie, 9188 Universität Tübingen, Auf der Morgenstelle 18, 72076 Tübingen, Germany; ‡ Institut für Physikalische und Theoretische Chemie, Universität Tübingen, Auf der Morgenstelle 18, 72076 Tübingen, Germany; § Institut für Anorganische Chemie, Universität Tübingen, Auf der Morgenstelle 18, 72076 Tübingen, Germany

## Abstract

Understanding the electronic structure of polycyclic aromatic compounds is of fundamental importance for their potential applications. The optoelectronic properties of shorter acenes such as tetracene and pentacene have been extensively studied with regard to excitation, emission, and nonlinear effects such as singlet fission. The longer homologues present a unique challenge due to their low stability both in the solid state and in solution. In this work, we synthesized persistent 6,8,15,17-tetrakis­((triisopropylsilyl)­ethynyl)­heptacene and investigated its photophysical properties as well as those of the parent heptacene. Our steady-state electronic absorption and emission experiments combined with transient absorption spectroscopy show that the Franck–Condon electric-dipole-forbidden (“dark”) transition to the 2^1^A_g_ singlet state is the lowest-energy excited state of heptacene. This contrasts with the optical properties of the well-known shorter acenes. Transient absorption data further suggest singlet fission or intersystem crossing as potential pathways to rapid population of the triplet state facilitated by the dark singlet state.

## Graphical abstract

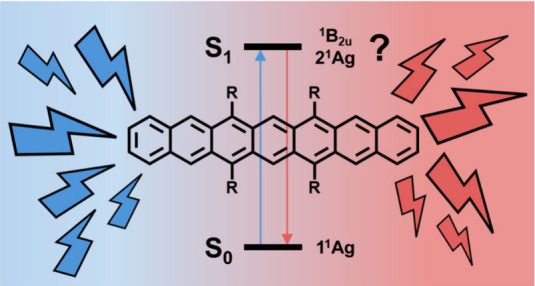

The electronic structure of polycyclic aromatic compounds is the key to their application as organic optoelectronic materials. A particularly important class of condensed polyaromatics are oligoacenes, as their study has driven development in singlet fission research over the past decade.^1–3^ The energies of the lowest-energy singlet and triplet states, S_1_ and T_1_, of the most important representatives, tetracene and pentacene, fulfill the energy requirement *E*(S_1_) ≈ 2*E*(T_1_) for singlet fission.^1,4–6^ Electronic excitation from the ground state to the S_1_ state (1^1^A_g_ → 1^1^B_2u_ transition) gives rise to a prominent band (p band according to Clar,^7^ ^1^L_a_ according to Platt^8^) in the absorption spectra of tetracene and pentacene.^9^

Increasing the conjugation length results in a bathochromic shift of the ^1^L_a_ band due to a decrease of the energy gap between the highest occupied molecular orbital (HOMO) and lowest unoccupied molecular orbital (LUMO).^4,10^ A decreasing HOMO–LUMO gap can result in a change of the character of the S_1_ state. For example, from octatetraene onward, polyenes have a lowest excited state that has the same symmetry as the ground state (^1^A_g_).^11–14^ According to computations using the particle–particle random phase approximation in combination with density functional theory (pp-RPA-B3LYP)^15^ and the multiconfiguration coupled-electron pair approximation (MC-CEPA),^16^ heptacene is the first acene in which, within the Franck–Condon approximation, the electric-dipole-forbidden 1^1^A_g_ → 2^1^A_g_ (“dark”) transition is lower in energy than the electric-dipole-allowed (“bright”) ^1^A_g_ → ^1^B_2u_ transition.^15,16^ The electronic absorption spectra of heptacene in solution, in a cryogenic argon matrix, and in organic glasses and polymers show a weak feature at lower energy than the ^1^L_a_ band that is absent in the shorter homologues.^9,16–21^ This feature, a shoulder in most cases and only in argon an individual new band,^9,16,20^ was tentatively associated with a forbidden transition in a previous study based on comparison with the computational data.^16^

We reasoned that the high kinetic stability of a heptacene derivative would be beneficial for the detailed optical spectroscopic investigations and opted for the synthesis of the persistent derivative 6,8,15,17-tetrakis­((triisopropylsilyl)­ethynyl)­heptacene (**TIPS4Hep**) (Scheme 1). During the course of our work, this compound was obtained by Bunz et al. using a different synthetic route.^22,23^ Here, by combining steady-state and transient absorption spectroscopy of **TIPS4Hep** and the parent heptacene (**Hep**), we show that the dark state 2^1^A_g_ becomes the lowest singlet excited state in the acene series. Unlike the conventional S_1_ state (^1^L_a_, 1^1^B_2u_), this dark state has a different leading electronic configuration (H^0^L^2^; H = HOMO, L = LUMO) compared to the T_1_ state (H^1^L^1^). This change in state order facilitates energy transfer from the dark state to an excited triplet state via intersystem crossing or singlet fission because of El-Sayed’s rule.

**Scheme 1:**
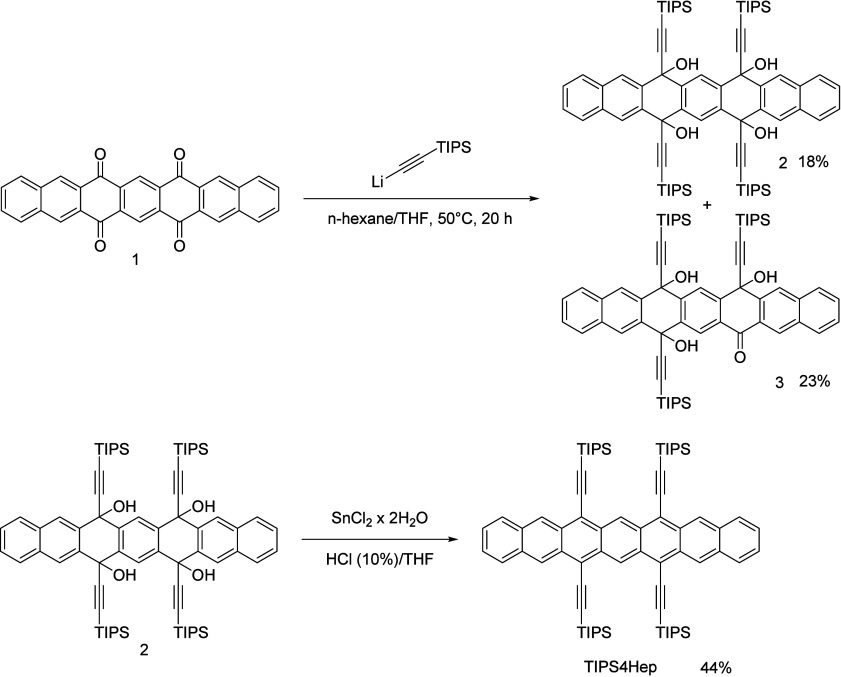
Synthesis of **TIPS4Hep** Starting from Heptacene-6,8,15,17-diquinone **1**

The synthesis of **TIPS4Hep** was performed using heptacene diquinone **1** (Scheme 1). The latter was synthesized following the route of Baxter et al.^24^ In addition to the analytical data given previously (IR, UV/vis, and EA),^24^ we achieved characterization by HR-APCI-MS as well as ^1^H NMR in D_2_SO_4_ (see the Supporting Information (SI)). Treatment of the diquinone with lithium triisopropylsilylacetylide at 50 °C not only gives the fourfold addition product tetrol **2** (18%) but also the threefold addition product triol **3** (23%). When this reaction is performed at room temperature, only threefold addition occurs. Tetrol **2** was further reduced with SnCl_2_·2H_2_O to the fully aromatic **TIPS4Hep** (Scheme 1). This reaction was performed in the absence of light in degassed solvents and gave the product in 44% isolated yield. **TIPS4Hep** is stable enough for chromatography to be performed over a short column in the dark, thus achieving the purity needed for detailed photophysical measurements.

**TIPS4Hep** was fully characterized by NMR, HRMS, and single-crystal X-ray crystallography. Bunz et al. recorded the ^1^H NMR spectrum in CDCl_3_ and justified the broad signals with the diradicaloid character of heptacene.^22^ We measured the ^1^H NMR spectrum in C_6_D_6_ and obtained sharp signals (see the SI). The broadening in CDCl_3_ likely stems from aggregation at the concentration required for NMR spectroscopy. We were able to measure a ^13^C NMR spectrum in C_6_D_6_ (see SI), while Bunz et al.^22^ stated that this was impossible due to the instability of **TIPS4Hep**. Unlike Bunz et al.,^22^ we could not detect an EPR signal in DCM, C_6_F_6_, or *n*-hexane. We could grow single crystals of **TIPS4Hep** suitable for single-crystal X-ray analysis (Figure S1, CCDC 2272243, *R* = 1.9%) by slow evaporation of a solution in DCM/*n*-hexane. The compound crystallized in space group *P*2_1_/*c*. Bunz et al. reported two crystal structures of this compound, one in space group *P*1̅ (CCDC 2254466, C_6_H_6_, *R* = 5.7%)^22^ and the other one also in *P*2_1_/*c* (CCDC 2254465, CHCl_3_/MeOH, *R* = 5.5%).^23^

The absorption spectrum of **TIPS4Hep** shows unusual features in the low-energy range compared to shorter acenes besides the known p band at 851 nm (A1), namely the shoulder at 887 nm (A2) and the weak band at 1010 nm (A3) (Figure 1). C_6_F_6_ proved to be suitable for our measurements, as it does not possess any absorption bands below 2000 nm, unlike other solvents with C–H bonds (Figures S8 and S9). Similar features were observed for **TIPS4Hep** by Bunz et al.^22^ as well as for other substituted heptacenes by Anthony et al.^18^ and Wudl et al.^19^ (Figures S4 and S5). Their origin was, however, not discussed in previous studies. TD-DFT calculations of the electronic absorption spectrum and the vibrational coupling for the parent heptacene cannot explain these peaks, as previously discussed (Figure S7).^16^

**Figure 1:**
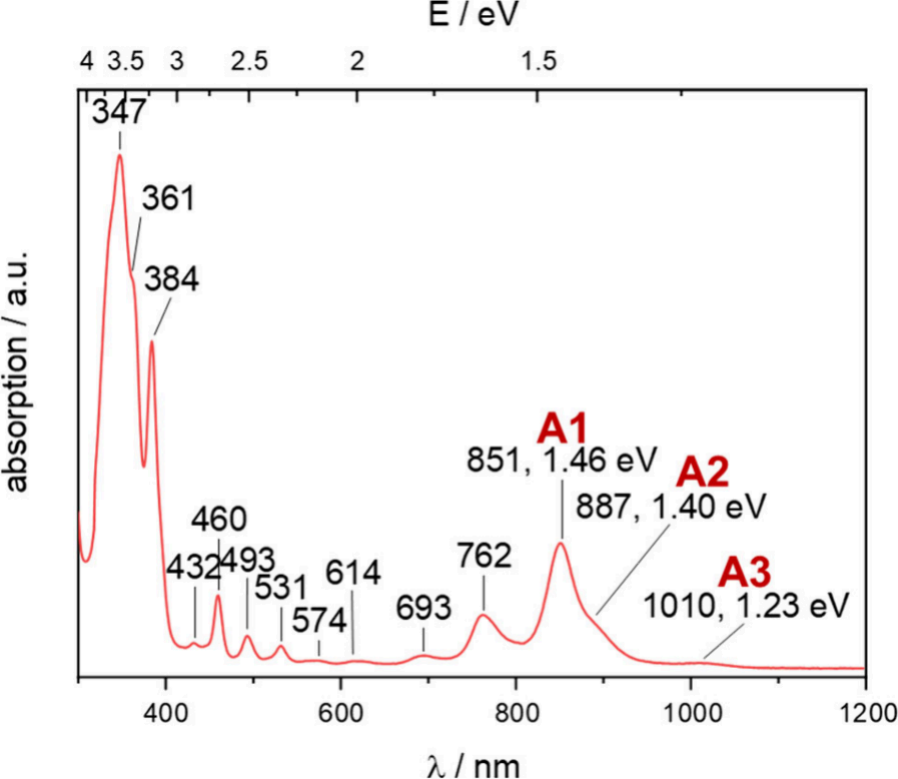
Absorption spectrum (1 × 10^–5^ M, rt) of **TIPS4Hep** in C_6_F_6_.

We performed a series of absorption spectroscopy experiments to confirm that the shoulder (887 nm, A2) as well as the 1010 nm band (A3) are due to **TIPS4Hep**. Measurements in solvents of different polarities revealed that A2 and A3 become more prominent with increasing polarity (Figure 2a). In *n*-hexane A3 was barely visible, whereas in 1:3 DCM/CH_2_I_2_ shoulder A2 and A3 are most prominent. The concentration-dependent measurements (Figure 2b) in the range 5 × 10^–6^ to 2 × 10^–4^ mol/L verified that neither transition is due to aggregation phenomena. Temperature-dependent measurements (Figure 2c) in toluene from +80 °C to −80 °C showed that with decreasing temperature both A3 and A2 become more prominent, ruling out a possible hot band. At lower temperature another shoulder of the second vibrational progression at 800 nm appears. Oxidation of **TIPS4Hep** with Ag­[Al­(OC­(CF_3_)_3_)_4_][Bibr ref25] (Figure 2d) resulted in a completely different spectrum, which fits into the series of monocationic acenes reported by Krossing et al.^26^ and is quite similar to that of the heptacene radical cation reported earlier under matrix isolation and gas-phase conditions.^20,27^ Hence, the latter experiment proves that the additional peaks are not due to traces of the radical cation. An EPR spectrum of this radical cation could be recorded (Figure S15) and is comparable to the EPR signals of a radical cation of sixfold-substituted heptacene reported by Bunz et al.^21^

**Figure 2:**
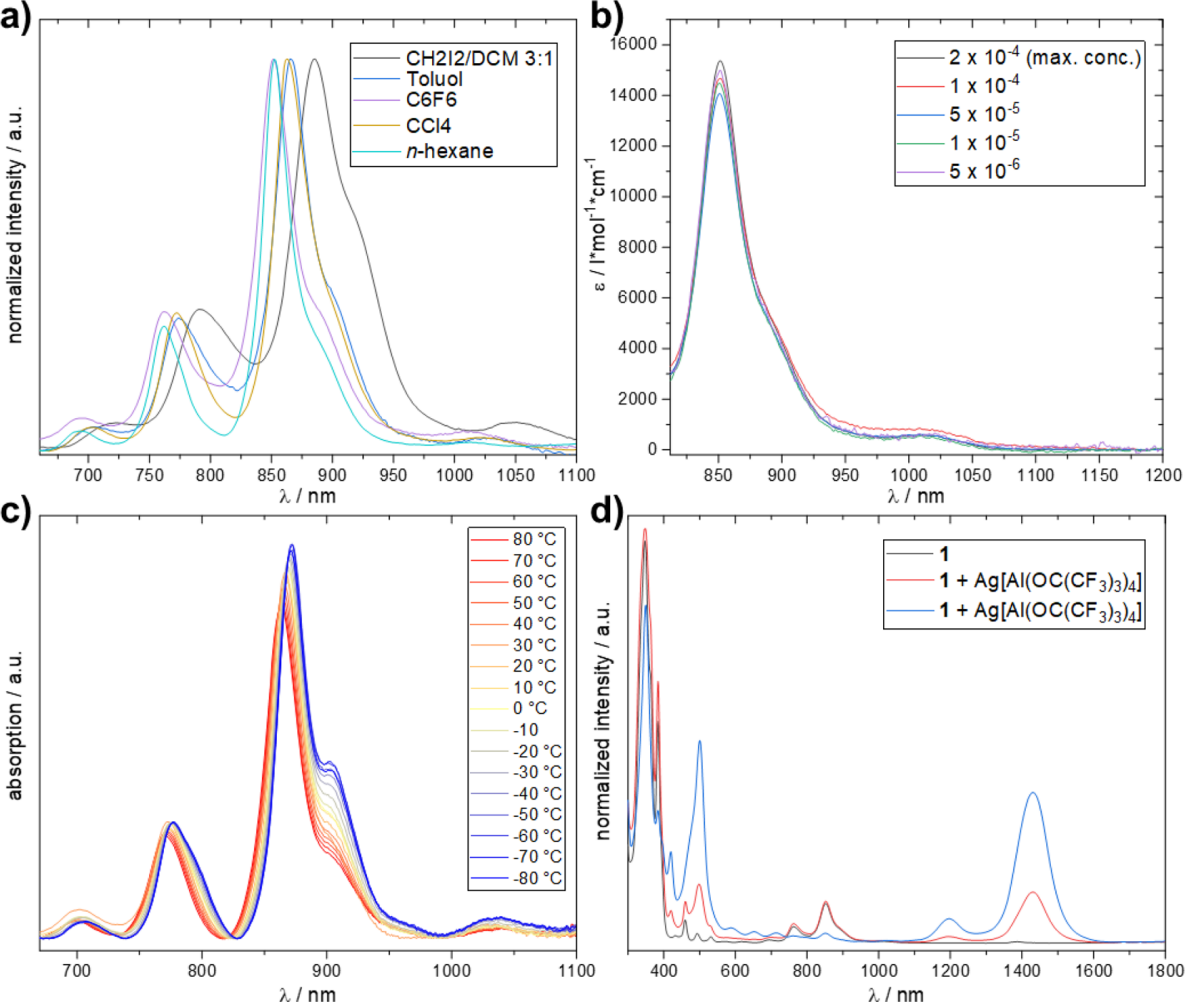
Absorption spectroscopy experiments: (a) solvent-dependent (1 × 10^–5^ M), (b) concentration-dependent (5 × 10^–6^ to 2 × 10^–4^ M, C_6_F_6_, rt), (c) temperature-dependent (toluene, 1 × 10^–5^ M, −80 to 80 °C in 10 °C steps), and (d) oxidation of **TIPS4Hep** with Ag­[Al­(OC­(CF_3_)_3_)_4_] (C_6_F_6_, 1 × 10^–5^ M, rt).

The shoulder A2 in the absorption spectrum of **TIPS4Hep** resembles features observed previously for **Hep** under cryogenic (Ar, 10 K) and room-temperature (PMMA) matrix isolation conditions and in solution (1-methylnaphthalene, 230 °C).^9,16,20^ In the solid argon spectrum of **Hep**, a distinct band with a maximum shifted by 730 cm^–1^ from the ^1^L_a_ maximum is observed, while in solution there is a shoulder shifted by about 650 cm^–1^ from the maximum to lower energies (see Figure S6).^16^ The shoulder in **TIPS4Hep** is similarly shifted in C_6_F_6_ by about 590 cm^–1^ from the maximum. This suggests that the carrier of A2 is related in **Hep** and **TIPS4Hep**. The weak maximum of **TIPS4Hep** at 1010 nm (A3, bathochromic shift of ∼1850 cm^–1^ with respect to the maximum), on the other hand, is not related to any known transition of **Hep**.

To investigate the possible origin of the A3 band of **TIPS4Hep** further, we employed fluorescence spectroscopy in different solvents. Characteristic for the fluorescence spectra of acenes are the small Stokes shift and the vibrational fine structure of the emission band that mirrors the absorption spectrum.^4^ The longest acene for which fluorescence spectra are known is hexacene.^4^ Fluorescence spectra of larger acenes are not available, to the best of our knowledge. We could obtain fluorescence spectra of **TIPS4Hep** in C_6_F_6_, toluene, and *n*-hexane (Figure 3a). CCl_4_ and C_2_Cl_4_ turned out to be unsuitable, as it appears that photoexcited heptacene can react with these solvents. This was shown by a red coloration of the initially almost colorless solution (Figure S10) and no detectable fluorescence. In all cases the strongest maxima of the fluorescence spectra were at very long wavelengths (C_6_F_6_, 1040 nm; toluene, 1064 nm; *n*-hexane, 1031 nm). The measured spectra looked very similar, showing consistency across the measurements in different solvents. In *n*-hexane (red), more features of the spectrum are resolved, as the shoulder at 1162 nm became more prominent compared to the other solvents and another shoulder at 1220 nm could be detected. Measurements in frozen solutions at 77 K (Figure 3b) revealed more features in toluene (1028, 1176 nm) and particularly in *n*-hexane (1019, 1064, 1158, and 1239 nm), as this solvent can form a Shpolskii matrix,^28–30^ which surrounds the molecule with a well-defined frozen solvent structure. The features of the corresponding excitation spectra largely follow the absorption spectra (Figure 3c,d). At higher energies, all main features are visible, but at longer wavelengths, the agreement is not as strong. Especially the band at 850 nm does not contribute to the emission as much as the less intense absorbing peak at approximately 760 nm. This possibly stems from effects of the gratings, which do not perform very well in these wavelength regions.

**Figure 3:**
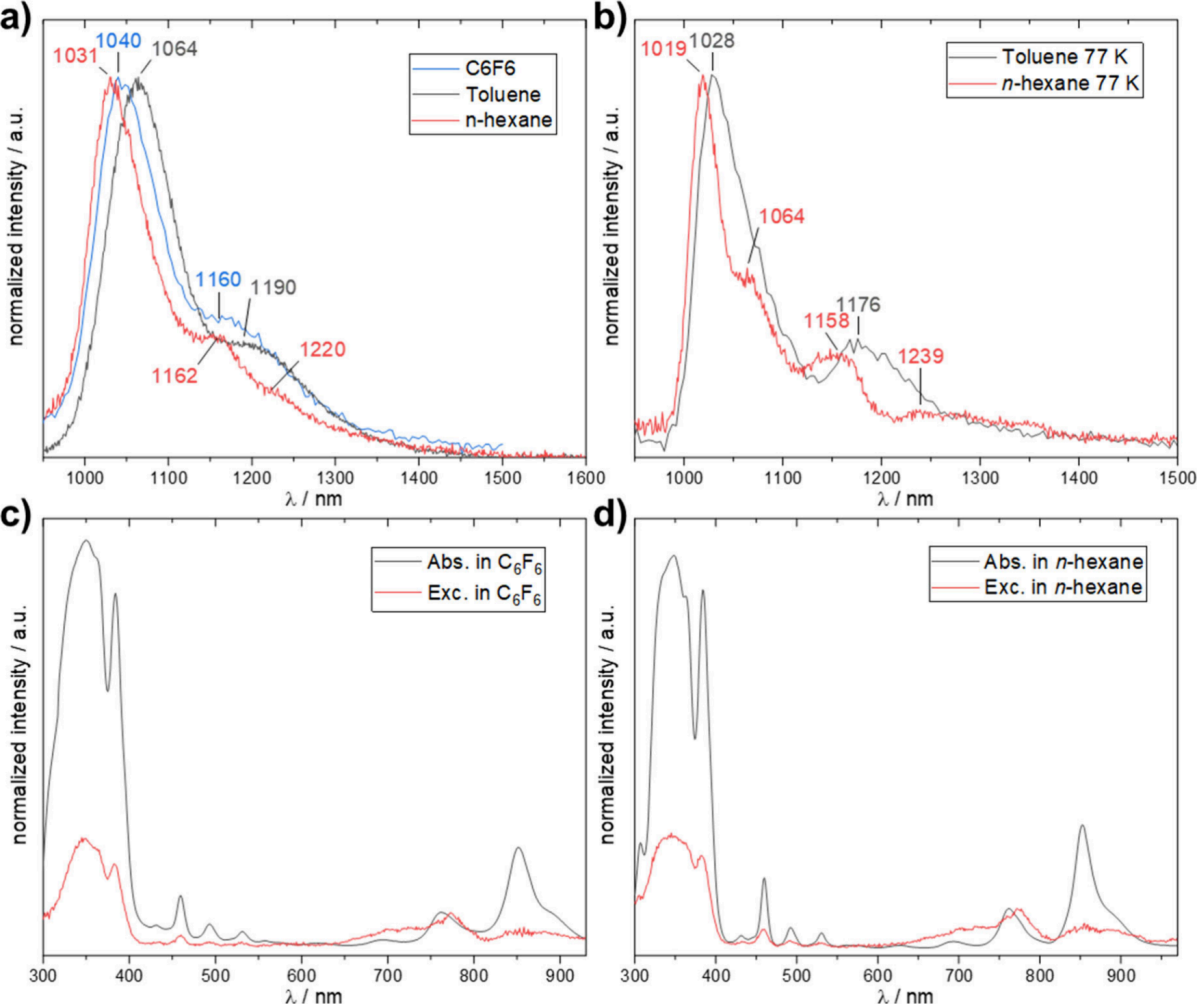
(a, b) Fluorescence spectra of heptacene in different solvents (1 × 10^–5^ M) with λ_exc_ = 350 nm at (a) room temperature and (b) 77 K. (c, d) Excitation spectra vs absorption spectra of **TIPS4Hep** in (c) *n*-hexane (λ_em_ = 1028 nm) and (d) in C_6_F_6_ (λ_em_ = 1040 nm).

To further support these measurements, we decided to take a closer look at parent **Hep**. Studying the photophysics of this molecule is quite challenging, as it can dimerize or polymerize in solution,^31^ although measurement in 1-methylnaphthalene at 230 °C allowed an absorption spectrum in solution to be recorded.^16^ A convenient alternative is to use a photoprecursor of **Hep** and a solid matrix, as has been shown by Neckers, Bettinger, and co-workers.^9,17,20^ In the present work, we used the monocarbonyl precursor **COHep** ^32^ (Figure 4), which is known to give **Hep** upon thermolysis in the solid state, as shown by Chow et al.^33^ The absorption spectrum of **Hep** in an argon matrix generated from **COHep** (Figure S14) is very similar to that obtained from the α-diketone precursor reported previously.^17,32^ Irradiation of the precursor **COHep** in a 2-methyltetrahydrofuran (MeTHF) glass at 77 K using a medium-pressure mercury vapor lamp and a dichroic mirror (350–450 nm) resulted in **Hep** (Figure 4a,b), as confirmed by characteristic signals of the heptacene π system in the 700–800 nm range.^9,16,17,20^

**Figure 4:**
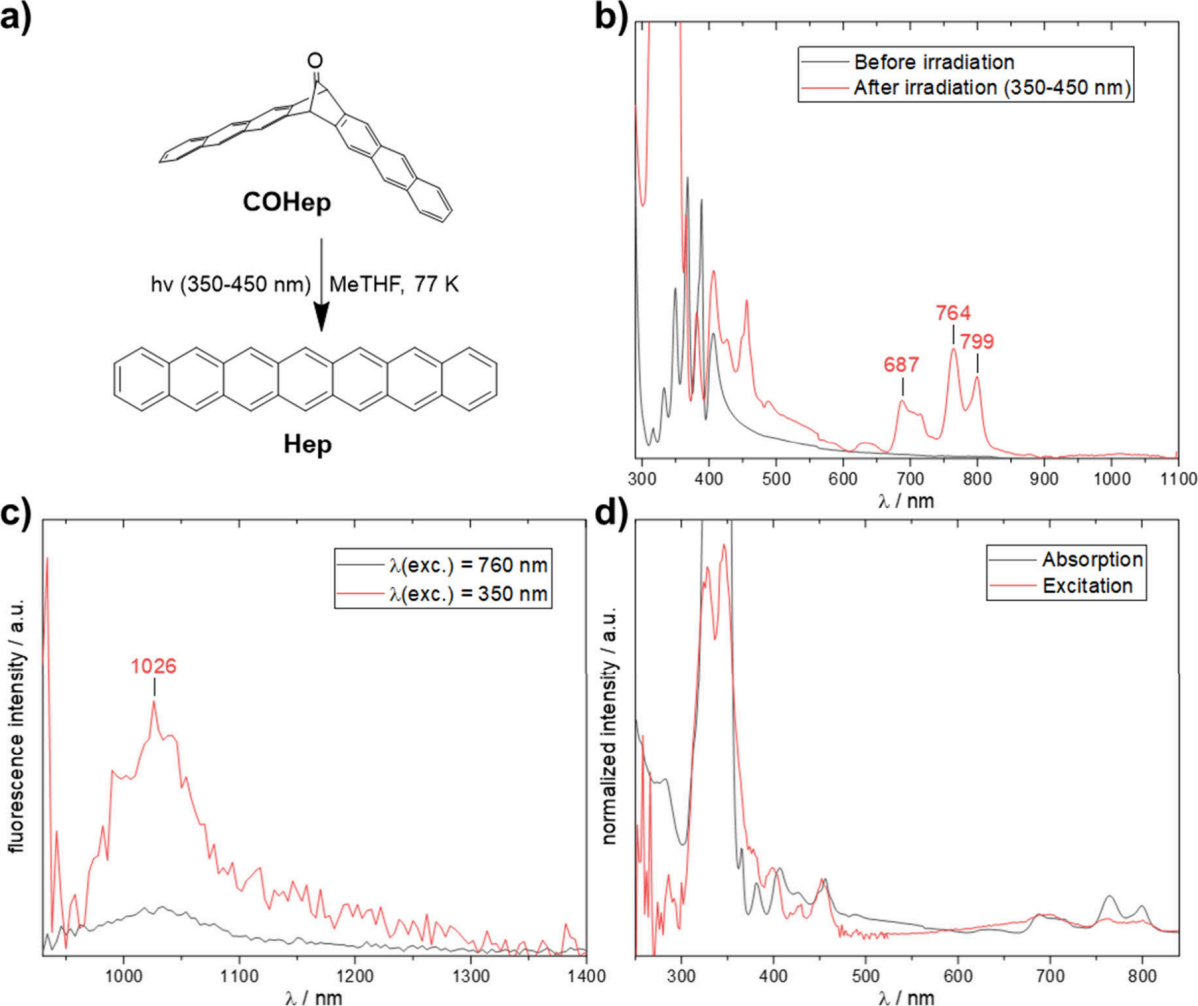
(a) Formation of **Hep** via monocarbonyl photoprecursor **COHep** in 2-MeTHF at 77 K and (b) the resulting absorption spectra before and after irradiation. (c) Emission spectrum of **Hep** in MeTHF at 77 K with λ_exc_ = 350, 760 nm. (d) Excitation spectrum (λ_em_ = 1030 nm) of **Hep** in MeTHF inside a J. Young tube at 77 K compared to the absorption of **Hep** inside a cuvette.

The fluorescence spectrum was measured under argon inside a J. Young tube in MeTHF at 77 K (for the absorption spectrum in a J. Young tube, see Figure S2), and a signal at approximately 1030 nm was the only signal that we could detect (Figure 4c). Comparison of the fluorescence excitation and absorption spectra (Figure 4d) shows some resemblance at short wavelengths, while at longer wavelengths discrepancies are apparent. This is consistent with the fluorescence excitation and absorption spectra of **TIPS4Hep** (Figure 3) discussed above. Emission at around 1030 nm would imply a very large Stokes shift if the longest-wavelength absorption at around 800 nm were considered as the emitting state, which seems highly unlikely in view of the typically very small Stokes shifts exhibited by shorter acenes.^4^

A plausible interpretation of the absorption and emission spectra of **Hep** and **TIPS4Hep** involves the 2^1^A_g_ state as the lowest-energy excited singlet state S_1_ of the heptacene framework. The lowest-energy excitation, 1^1^A_g_ → 2^1^A_g_ (S_0_ → S_1_), is electric-dipole-forbidden within the Franck–Condon approximation in this case and not detectable for **Hep** but appears as a low-intensity band (A3) at 1010 nm for the lower-symmetry **TIPS4Hep**. The energy difference between the p band maximum A1 (1^1^A_g_ → 1^1^B_2u_) at 852 nm and A3 in *n*-hexane is 0.23 eV (0.23 eV in C_6_F_6_, 0.22 eV in toluene, and 0.22 eV in 1:3 DCM/CH_2_I_2_), which is in reasonable agreement with the energy gap computed previously for these states (0.17 eV).^16^ Assuming a similar energy gap between 1^1^B_2u_ and 2^1^A_g_ for **Hep** as for **TIPS4Hep**, we would expect the 1^1^A_g_ → 2^1^A_g_ absorption to be located at around 932 nm for **Hep**. Emission spectroscopy can reveal the 2^1^A_g_ → 1^1^A_g_ transition due to its sensitivity for both **Hep** and **TIPS4Hep**. The Stokes shift of 0.02 eV (181 cm^–1^) for **TIPS4Hep** in *n*-hexane is reasonably small, as typically observed for acenes. The predicted 1^1^A_g_ → 2^1^A_g_ absorption of **Hep** at 932 nm could correspond to a Stokes shift of 0.13 eV (1021 cm^–1^), which appears to be too large. Adding the Stokes shift of **TIPS4Hep** to the fluorescence signal of **Hep** would lead to a predicted 1^1^A_g_ → 2^1^A_g_ absorption at 1016 nm. Thus, we would expect the missing transition of **Hep** to be between 932 and 1016 nm. The vibrational fine structure of the fluorescence spectrum of **TIPS4Hep** indicates that the 2^1^A_g_ → 1^1^A_g_ transition goes along with vibrational excitation, and the same must be expected for the 1^1^A_g_ → 2^1^A_g_ absorption. The first vibrational band in the fluorescence spectrum of **TIPS4Hep** in *n*-hexane at room temperature at 1162 nm is shifted by 1094 cm^–1^ (C_6_F_6_, 995 cm^–1^; *n*-hexane (77 K), 1178 cm^–1^; toluene, 1022 cm^–1^; toluene (77 K), 1225 cm^–1^) from the fluorescence origin (Figure 3a), and assuming a similar shift in the 1^1^A_g_ → 2^1^A_g_ absorption, the shoulder (A2) at 890 nm (C_6_F_6_, 887 nm; toluene, 898 nm) is due to the vibrational feature of this transition (expected at 911 nm with these numbers; C_6_F_6_, 919 nm; toluene, 930 nm; Figure S3). This gains intensity from the nearby 1^1^A_g_ → 1^1^B_2u_ transition. The low-temperature absorption spectrum in toluene in Figure 3c shows another shoulder at the first p vibrational band. Between this shoulder, the main shoulder A2 and the 1010 nm band A3, an energy gap of 0.18 eV (1450 cm^–1^) can be determined, which is a further indication of a vibrational progression. Likewise, the weak band at 768 nm in the matrix spectra of **Hep** is due to a vibrational progression of the 1^1^A_g_ → 2^1^A_1g_ transition.^9,16^

We performed transient absorption spectroscopy (TAS) of a 50 μM **TIPS4Hep** solution in C_6_F_6_ under argon to further corroborate our hypothesis of a 2^1^A_g_ singlet excited state. TAS probes the temporal evolution of the excited state by determining the differential absorbance Δ*A*, which is the difference in absorbance between the excited and ground states. We pumped **TIPS4Hep** with 90 fs laser pulses at 900 nm to selectively target the A3 transition and monitor the excited-state absorption within a time window of 200 fs to 20 μs. Experiments at short time scales (<2 ns) were carried out with 0.9 μJ/pulse. To enhance the visibility of the weaker signals at long delay times, we display here the spectra obtained with 8 μJ/pulse but note that similar results (with less favorable signal-to-noise ratio) were observed with 0.9 μJ/pulse (see the SI for more details).

Figure 5a depicts a superimposed 2D hyperspectrum as a function of the pump–probe delay time and Δ*A*, which is color-coded on the right. The white patches indicate spectral regions that cannot be probed due to scattered pump or weak probe light. Detailed Δ*A* spectra at selected delay times below 400 ps are shown in Figures 5b and S11. We find bands with negative Δ*A* centered at 350, 383, 850, and 1020 nm, which are consistent with the steady-state absorbance shown in red above the 2D spectrum in Figures 5a and 1. Negative Δ*A* values indicate ground-state bleaching (GSB) due to a partial depopulation of the ground state as well as Pauli blocking (S_0_ →̷ S_*n*_, *n* ≠ 0) due to partial filling of an excited state. The fact that the β band at 350 nm and the p band at 850 nm (A1) both exhibit GSB under 900 nm excitation is a strong indication that the band at 1020 nm (A3) involves the 1^1^A_g_ ground state. In addition, we observe bands with positive Δ*A*, indicating excited-state absorbance (ESA) (S_*n*_ → S_*n*+*x*_, *n*, *x* ≠ 0), which is most prominent at 620 nm and some features around 500 nm. In the NIR, weak ESA around 1120, 1290, and 1500 nm emerges, showing a possible vibrational progression (compare Figure S11).

**Figure 5:**
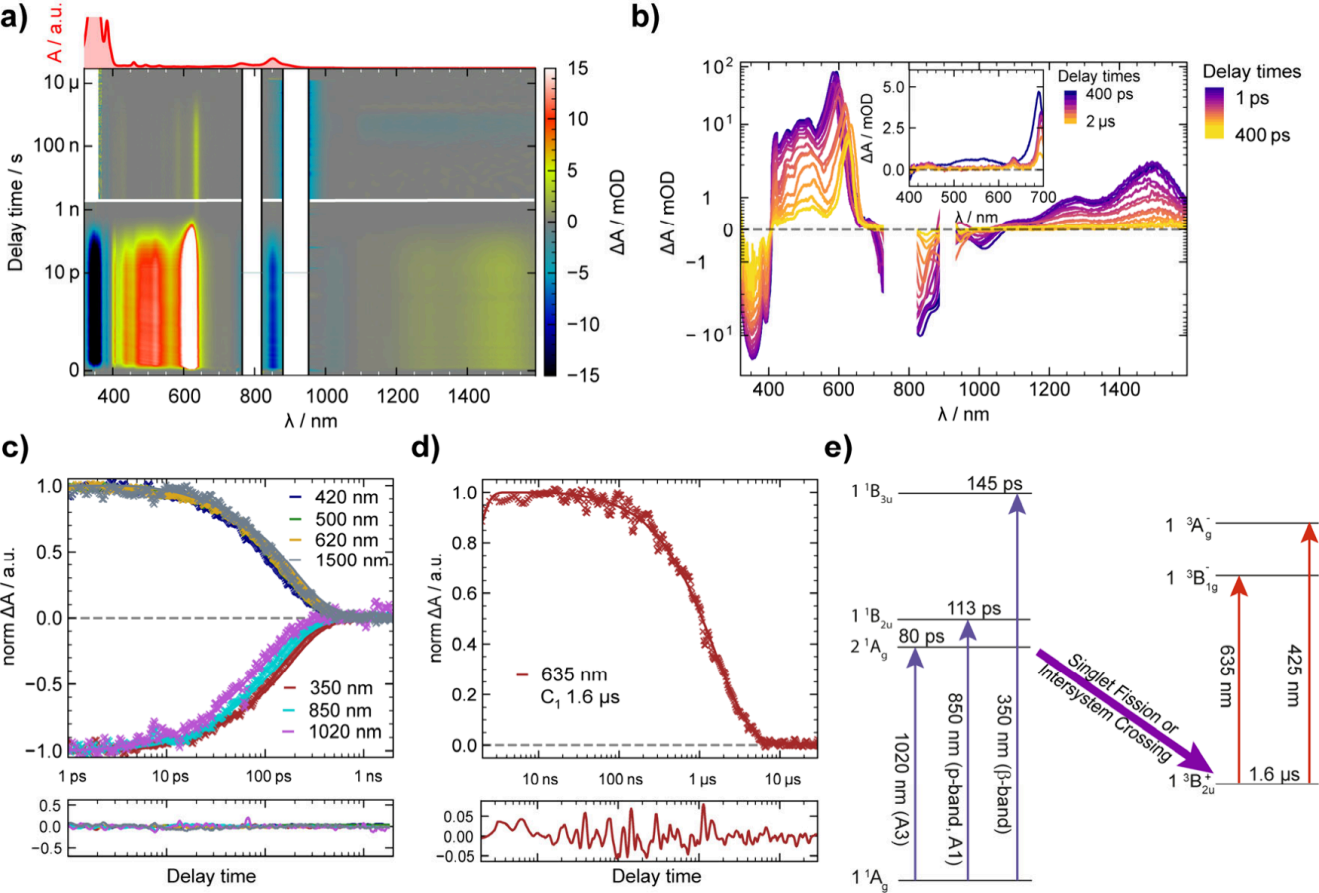
Transient absorption spectra of **TIPS4Hep** in C_6_F_6_ (λ_exc_ = 900 nm). (a) Superimposed 2D TA spectra consisting of fs TAS up to 2 ns (0.9 μJ/pulse) and ns TAS (8 μJ/pulse) up to 20 μs. A steady-state absorbance spectrum is shown in red at the top. (b) Δ*A* spectra of (a) at selected delay times. In the inset, the transition from ps to μs TAS is shown. (c, d) Kinetic traces and their corresponding fits at selected wavelengths. The residuals of the fits are displayed below the graphs. (e) Schematics of all relevant states, transition wavelengths, and lifetimes extracted from the TAS experiments. Blue and red arrows indicate ground-state bleaches and excited-state absorption, respectively. All term symbols are according to Chakraborty and Shukla.^34^

We fitted the kinetics of all prominent GSB and ESA features in Figure 5c and found that most of the transient signals decay monoexponentially within 130 to 160 ps. Across the full probe spectrum, we observe no significant kinetics within the first picosecond other than the ∼400 fs population rise imposed by the instrument response and very weak residual coherent artifacts. The lifetimes of the three prominent GSB signals are 145 ps at 350 nm (red, β-band), 113 ps for the p band (cyan, A1), and 80 ps for A3 (purple). These overall comparable magnitudes are supporting evidence that the A3 transition belongs to **TIPS4Hep**. The small but significant differences in the lifetimes of the three transitions can be rationalized in terms of a shared ground state but different excited states. This is consistent with our attribution of the A3 band to a 1^1^A_g_ → 2^1^A_g_ transition. With respect to the ESA kinetics, the most notable finding is that the band at 635 nm exhibits complex kinetics with a near-monoexponential decay to 3% of the peak Δ*A* value, where it remains as a residual, constant ESA for >8 ns (see Figure S12).

In Figure 5d, we display the further monoexponential decay of this residual ESA and determine a lifetime of 1.6 μs. We note additional ESA signals with such long lifetimes in Figure 5a at 425, 585, and 635 nm, which are plotted in more detail in the inset of Figure 5b. We argue that these features are evidence for a populated triplet state and assign the ESA at 635 and 425 nm to the 1^3^B_2u_^+^ → 1^3^B_1g_^–^ and 1^3^B_2u_^+^ → 1^3^A_g_^–^ transitions, respectively. We rationalize this with two previous findings: heptacene prepared in situ by the photodecarbonylation of α-diketones revealed a triplet with a lifetime of 11 μs featuring a sharp ESA centered at 580 nm (2.14 eV) and a broad band between 400 and 500 nm.^35^ ESA from the 1^3^B_2u_ triplet in heptacene has been calculated with a prominent transition at 580 nm (1^3^B_2u_^+^ → 1^3^B_1g_^–^) and a weaker transition at 393 nm (1^3^B_2u_^+^ → 1^3^A_g_^–^).^34^ These values are in reasonable agreement with our experimental results for **TIPS4Hep**, accepting a shift of ∼0.2 eV to lower energies for both transitions, which may be the effect of the TIPS-ethynyl substituents. In line with this, our computed absorption spectrum (Figure S13) of T_1_-**TIPS4Hep** obtained using TD-UDFT (UB3LYP/6-311+G**//M06-2X/def2-SVP) shows two main transitions with high oscillator strengths at 703 nm (*f* = 0.85) and 457 nm (*f* = 0.14), that is, at lower energies than the computational results for heptacene at the same level (see the SI for more details). The unassigned third transition at 585 nm can be regarded as a vibrational progression of the transition at 635 nm.

We conclude by discussing the population mechanism of the T_1_ state in **TIPS4Hep** based on our TAS data. We argue that the most likely pathway is via singlet fission (SF) or intersystem crossing (ISC) of the 2^1^A_g_ singlet. This would also explain why the GSB of the 1^1^A_g_ → 2^1^A_g_ transition has the shortest lifetime, as it would decay due to relaxation to S_0_ and T_1_ simultaneously. An involvement of the 1^1^B_2u_ state is unlikely for three reasons: (1) for this state to be populated with a 900 nm pump pulse, two-photon absorption would be required, which is unlikely with the 0.9 μJ/pulse utilized by us; (2) the ISC from this state is expected to be slow due to El-Sayed’s rule, which is incompatible with the competition with the rather fast decay of the 1^1^B_2u_ state (113 ps); (3) SF of the 1^1^B_2u_ state occurs on short enough time scales for TIPS-ethynyl-pentacene derivatives, where the ideal scenario of *E*(S_1_) = *E*(^1^(TT)) is met almost perfectly.^36,37^ For higher acenes, however, SF is increasingly exothermic, which greatly decreases its efficiency and favors triplet pair recombination before the free T_1_ state is formed.^38^

Assuming a similar energy for the 1^3^B_2u_ state in **TIPS4Hep** as the 0.5–0.6 eV calculated for **Hep**,^15,16^ SF should be much more favorable from the 2^1^A_g_ state (1.2 eV). We note that SF typically requires concentrations larger than 4 × 10^–4^ M,^36^ which is slightly more than the 5 × 10^–5^ M used by us. A possible explanation could be the temporal formation of small aggregates in solution.

Alternatively, the T_1_ state may be populated from the 2^1^A_g_ state via fast ISC, since it is favored by El-Sayed’s rule. Moreover, organic π radicals have shown ISC lifetimes on the order of picoseconds due to radical enhanced intersystem crossing (REISC).^39–41^ Heptacene is known for its biradical character,^15^ and this property could render ISC competitive to the S_0_ relaxation with τ = 80 ps.

We summarize our TAS results concerning the nature of all observed transitions, their lifetimes, the two coexisting spin systems, and the pathway of transition between them in Figure 5e.

In conclusion, we have developed an alternative synthesis for **TIPS4Hep** and conducted a comprehensive photophysical study using steady-state and transient absorption spectroscopy. Comparing these results with complementary spectroscopic data on the less stable parent **Hep** suggests that **TIPS4Hep** is a suitable proxy to understand the electronic structure and excited-state dynamics of **Hep**. Its absorption spectrum reveals a weak band at 1010 nm, assigned to a forbidden 1^1^A_g_ → 2^1^A_g_ transition that is partially allowed due to vibronic coupling and proximity to allowed transitions. Similar spectral features are observed for **Hep**, although its higher symmetry renders the 1^1^A_g_ → 2^1^A_g_ origin unobservable. In accordance with computational spectroscopy, we assign this dark excited state to the S_1_ state of heptacene. We show that the dark state relaxes within 80 ps to a long-lived triplet state, and we discuss singlet fission and intersystem crossing as the most likely mechanisms for this action.

## Supplementary Material



## Data Availability

The data underlying this study are openly available in RADAR4Chem at DOI: 10.22000/j80kn6cses15pn7x.

## Notes

Synthesis of all compounds and their ^1^H NMR, ^13^C NMR, and HRMS spectra; computational and additional experimental details for absorption, fluorescence, and transient absorption spectroscopy; Cartesian coordinates; absorption spectra from other publications (PDF)
