# Supplementary material for: The Lowest Excited State of Heptacenes Is Dark
Source: J Phys Chem Lett. 2025 Sep 3;16(36):9479–87. doi: 10.1021/acs.jpclett.5c01314 (PMC12434725; doi:10.1021/acs.jpclett.5c01314)
Supplement: Supplementary file 1 [file jz5c01314_si_001.pdf]

Supporting Information

The Lowest Excited State of Heptacenes Is Dark

Johannes Schöntag[a], Philipp Frech[b], Kathrin Zwettler[a], Navid Fardan[a], Ankit Somani[a], Wolfgang Leis[c], Markus Ströbele[c], Michael Seitz[c], Marcus Scheele[b]*, Holger F. Bettinger*[a]

[a] Institut für Organische Chemie, Universität Tübingen, Auf der Morgenstelle 18, 72076 Tübingen, Germany

E-mail: holger.bettinger@uni-tuebingen.de

[b] Institut für Physikalische und Theoretische Chemie, Universität Tübingen, Auf der Morgenstelle 18, 72076 Tübingen, Germany

E-mail: marcus.scheele@uni-tuebingen.de

[c] Institut für Anorganische Chemie, Universität Tübingen, Auf der Morgenstelle 18, 72076 Tübingen, Germany

Table of contents

General 2

Synthesis 3

Heptacene-6,8,15,17-diquinone (1) 3

Tetra-6,8,15,17-TIPS-ethinyl heptacene (Tetrol 2) 3

Tri-8,15,17-TIPS-ethinyl heptacene-6-one (Triol 3) 4

Tetra-6,8,15,17-TIPS-ethynyl heptacene (TIPS4Hep) 4

Monocarbonyl heptacene COHep 5

Crystallography 6

Tetra-6,8,15,17-TIPS-ethinyl heptacene (TIPS4Hep) 6

Additional Information 7

Literature known spectra of heptacene derivatives 8

Steady state absorption spectroscopy 10

Solvents in the Vis and NIR: 10

Fluorescence spectroscopy 12
Transient absorption spectroscopy (TAS) 13
Matrix Isolation 16
Experimental Details 16
Results 16
EPR 17
Computations 18
TD-UDFT 18
Electronic absorption spectrum of T_1 of TIPS4Hep 18
Experimental spectra 22
NMR 22
Mass spectrometry 26
References 30

General

All reactions were run under inert gas (Ar) conditions. THF and *n*-hexane were taken from a SPS-800 from *MBraun*. Chemicals were purchased from *Acros*, *Sigma-Aldrich*, *TCI Chemicals* and *fluorochem* and used without further purification. NMR spectra were recorded on a *BRUKER Avance 400* and a *Bruker Avance 700* at 400/700 MHz (^{1}H NMR) and 100/176 MHz (^{13}C NMR). Chemical shifts for ^{1}H NMR spectra are reported in ppm relative to TMS using the residual signals of chloroform-d (δ 7.26) and benzene-d6 (δ 7.16) as reference. Chemical shifts for ^{13}C NMR spectra are reported in ppm relative to TMS using the signal of chloroform-d (δ 77.00) and benzene-d6 (δ 128.1) as reference. Mass spectra with high resolution were recorded on a maXis-4G-spectrometer from *Bruker Daltonics* (ESI or APCI) with a TOF analyzer.

Synthesis

Heptacene-6,8,15,17-diquinone (1)

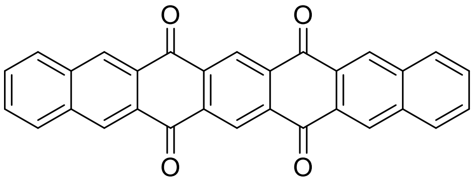

Heptacene-6,8,15,17-diquinone was synthesized following Baxter *et al.*[1]

HRMS (APCI): $[M+H]^+$ calculated m/z 439.09649, measured m/z 439.09638

^{1}H NMR (400.16 MHz, D_2SO_4): δ (ppm) 9.46 (s, 2H, H7), 9.18 (s, 4H, H5), 8.34-8.25 (m, 4H, H1), 8.06-7.98 (m, 4H, H2).

Tetra-6,8,15,17-TIPS-ethinyl heptacene (Tetrol 2)

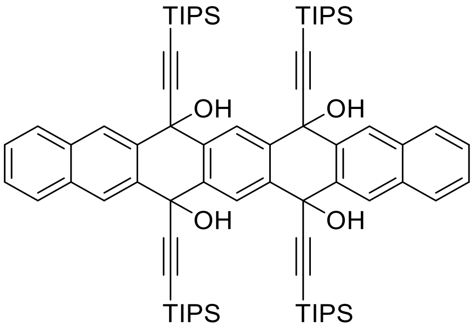

(Triisopropylsilyl)acetylene (333 µL, 1.48 mmol, 13 eq.) was dissolved in 12 mL dry *n*-hexane and *n*-BuLi (547 µL (2.5 M in *n*-hexane), 1.37 mmol, 12 eq.) was slowly added at rt. After stirring for 1 h, 0.5 mL dry THF was added and afterwards heptacene-6,8,15,17-diquinone (50.0 mg, 114 µM, 1 eq.). The mixture was heated and stirred at 50 °C for 20 h. The reaction was quenched with sat. aq. NH_4Cl, the phases were separated, and the organic phase dried over $MgSO_4$. After removing the solvent, the residue was purified by column chromatography (DCM/EtOAC 19:1, R_f = 0.3). Tetrol **2** was isolated as a light brown solid (24 mg, 20.5 µM, 18%) that was not completely pure, but of sufficient quality for the next step.

^{1}H NMR (400.16 MHz, $CDCl_3$): δ (ppm) 8.74 (s, 2H, H7), 8.60 (s, 4H, H5), 7.89-7.87 (m, 4H, H1), 7.55-7.51 (m, 4H, H2), 4.21 (br, 4H, O*H*), 1.09-1.04 (m, 84H, $(CH_3)_2CHSi$).

^{13}C NMR (176.09 MHz, C_6D_6): δ (ppm) 138.5, 135.8, 133.3, 128.2, 127.2, 126.9, 126.4, 107.9 (Ar-*C*-C-TIPS), 90.5 (Ar-*C*-C-TIPS), 69.3 (*C*-OH), 18.9 ($(CH_3)_2CHSi$), 18.8 ($(CH_3)_2CHSi$), 11.5 ($(CH_3)_2CHSi$)

HRMS (ESI): $[M+Na]^+$ calculated m/z 1189.67474, measured m/z 1189.67326

As a side product threefold substitution leads to triol **3** which was also isolated.

Tri-8,15,17-TIPS-ethinyl heptacene-6-one (Triol 3)

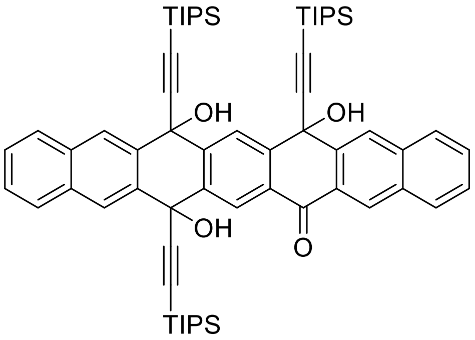

Obtained as by-product in the synthesis of tetra-6,8,15,17-TIPS-ethinyl heptacene (DCM, R_f = 0.2, 23%).

^{1}H NMR (400.16 MHz, 298.15 K, CDCl3]: δ (ppm) 9.10 (s, 1H), 9.00 (s, 1H), 8.87 (s, 1H), 8.70 (s, 1H), 8.66 (2s, 2H), 8.03-8.01 (m, 1H), 7.96-7.92 (m, 3H), 7.64-7.60 (m, 1H), 7.58-7.51 (m, 3H), 3.26 (br, s, 1H, OH), 3.17 (br, s, 1H, OH), 2.97 (br, s, 1H, OH), 1.12-1.05 (m, 63H, (C*H*3)2C*H*Si).

^{13}C NMR (176.09 MHz, C_6D_6): δ (ppm) 182.8, 144.7, 139.5, 139.3, 136.0, 135.8, 135.4, 133.4, 133.4, 132.9, 130.4, 130.0, 129.5, 129.1, 128.4, 128.3 (2C), 128.1, 127.5, 127.4, 127.1 (2C), 127.0, 126.6, 126.6, 126.2, 109.2 (2C), 108.7, 90.6, 90.0, 89.3, 69.2, 69.0, 67.2, 18.8, 11.4, 11.3

HRMS (ESI): $[M+Na]^+$ calculated m/z 1007.52566, measured m/z 1007.52548

Tetra-6,8,15,17-TIPS-ethynyl heptacene (TIPS4Hep)

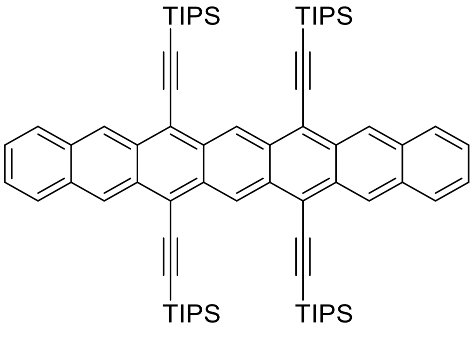

Tetra-6,8,15,17-TIPS-ethynyl heptacene tetrole **2** (48 mg, 41.1 µM, 1 eq.) was dissolved in degassed THF (10 mL). Degassed (Ar bubbled) aq. HCl (10%, 0.5 mL) and $SnCl_2 \cdot 2H_2O$ (186

mg, 824 μM, 20 eq.) was added and the reaction vessel was covered with aluminum foil. The mixture was stirred at 30 °C for 3 h. Argon was bubbled through the solution while the vessel is open to remove all the solvent in the absence of oxygen. Then a mixture of *n*-hexane/DCM (9:1) was added and the mixture poured onto a thick silica plug (7 cm height) in the dark. More solvent (*n*-hexane/DCM (9:1)) was added (400 mL) and it was quickly pushed through the plug via vacuum into an aluminum foil covered round bottom flask. The solvent was quickly removed on a rotary evaporator and the resulting light brown solid was quickly stored under Ar (19.9 mg, 44%).

^{1}H NMR (700.29 MHz, C_6D_6): δ (ppm) 10.14 (s, 2H, H7), 9.55 (s, 4H, H5), 8.00-7.99 (m, 4H, H1), 7.14-7.12 (m, 4H, H2), 1.46-1.45 (m, 84H, (C*H*$_3$)$_2$C*H*Si).

^{13}C NMR (176.09 MHz, C_6D_6): δ (ppm) 133.5, 132.8, 131.0, 128.9 (C1), 127.3 (C5), 126.7 (C7), 126.7 (C2), 119.2 (C6), 109.2 (Ar-C-*C*-TIPS), 106.5 (Ar-*C*-C-TIPS), 19.4 ((*C*H$_3$)$_2$CHSi), 12.5 ((*C*H$_3$)$_2$*C*HSi)

HRMS (APCI): $[M+H]^+$ calculated m/z 1099.68184, measured m/z 1099.68336

Monocarbonyl heptacene COHep

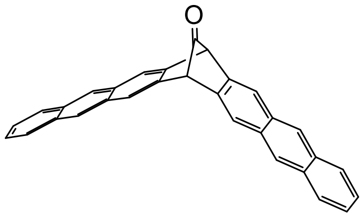

The synthesis of heptacene mono carbonyl precursor **COHep** was performed following the route of Gourdon *et al.*[2] The ^{1}H NMR spectrum was consistent with that reported by Gourdon *et al.*[2]

Crystallography

Tetra-6,8,15,17-TIPS-ethinyl heptacene (TIPS4Hep)

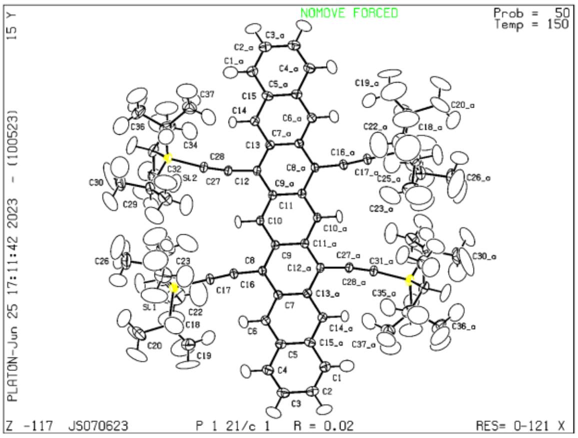

Formula	$C_{74}H_{98}Si_4$
CCDC	2272243
$D_{calc.}$/ g cm^{-3}	1.083
μ/mm^{-1}	1.103
Formula Weight	1099.944
Colour	clear colourless
Shape	block-shaped
Size/mm^3	0.19×0.19×0.15
T/K	150.01(10)
Crystal System	monoclinic
Space Group	$P2_1/c$
a/Å	16.0576(1)
b/Å	15.3073(1)
c/Å	14.9738(1)
α/°	90
β/°	113.644(1)
γ/°	90
V/Å^3	3371.57(5)
Z	2
Z'	0.5
Wavelength/Å	1.54184
Radiation type	Cu K$_\alpha$
Θ_{min}/°	3.00
Θ_{max}/°	79.94
Measured Refl's.	167341
Indep't Refl's	7360
Refl's I≥2 σ(I)	7215
R_{int}	0.0151
Parameters	794
Restraints	0
Largest Peak	0.2239
Deepest Hole	-0.2160
GooF	1.0773
wR_2 (all data)	0.0472
wR_2	0.0470
R_1 (all data)	0.0196
R_1	0.0192

Additional Information

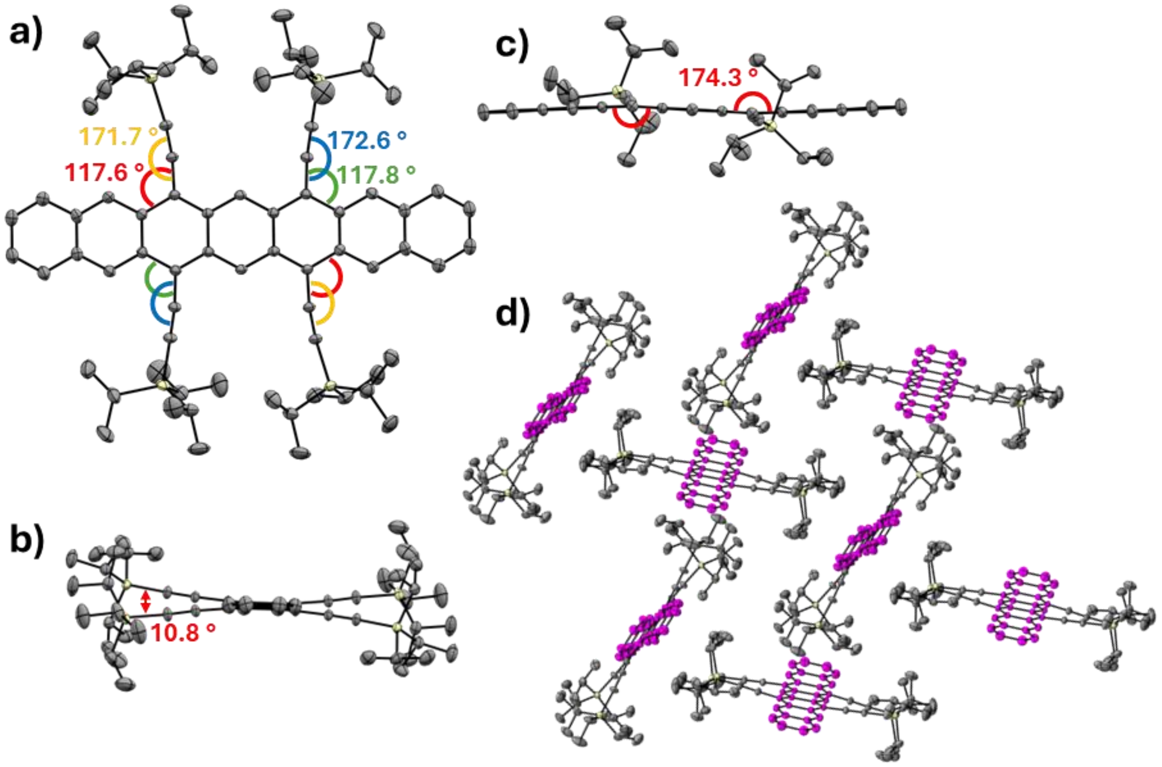

Figure S1. a) Molecular structure of **TIPS4Hep** in the solid state, b) and c) side view to emphasize the steric demand of the TIPS groups and the resulting distortion, d) herringbone packing of **TIPS4He**p. Anisotropic displacement parameters are depicted at the 50% probability level, hydrogen atoms are omitted for clarity.

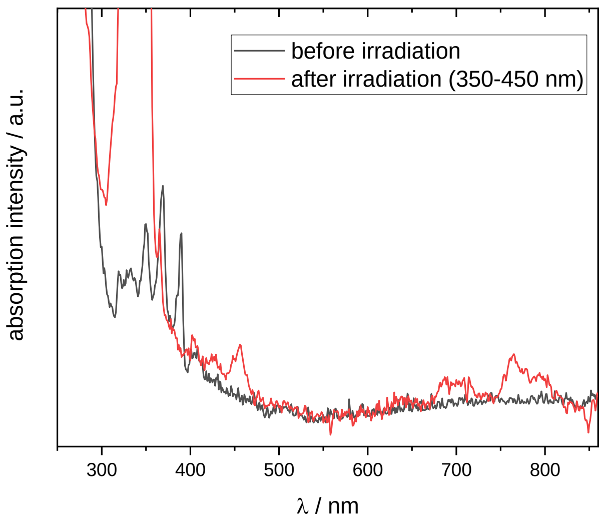

Figure S2. Absorption spectrum of the formation of heptacene **Hep** via mono carbonyl photo precursor **COHep** in 2-MeTHF at 77 K inside a J.-Young tube.

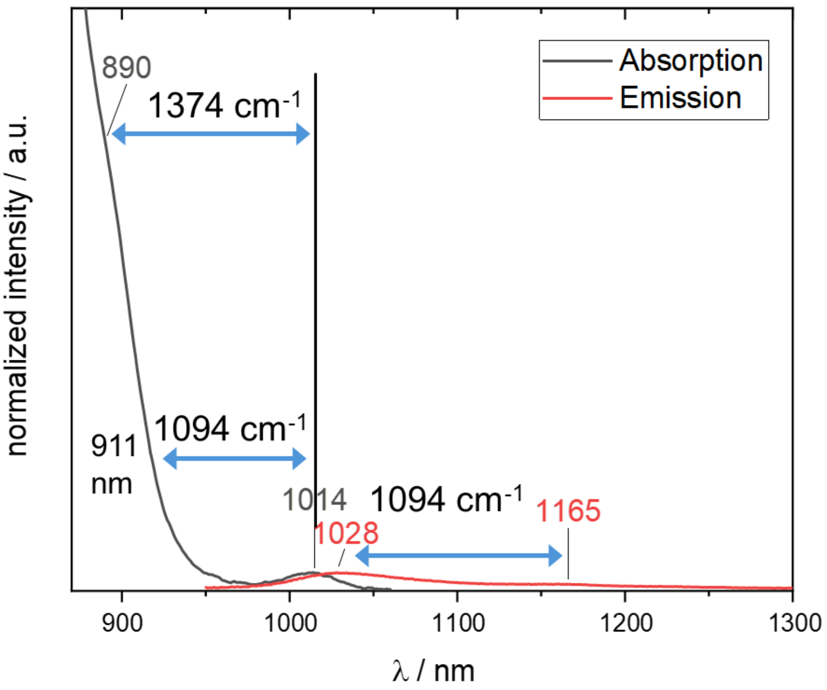

Figure S3. Distances of vibrational progression of absorption and emission of **TIPS4Hep** in *n*-hexane compared to each other.

Literature known spectra of heptacene derivatives

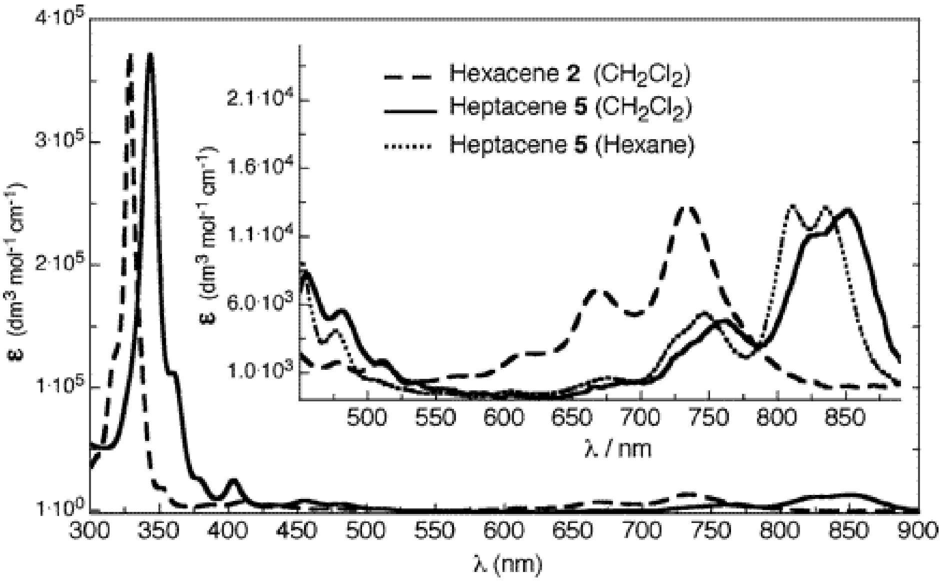

Figure S4. Absorption spectra of 7,16-silylethinylheptacene. Reprinted with permission from J. Am. Chem. Soc. 2005, 127, 22, 8028–8029.[3] Copyright 2005 American Chemical Society.

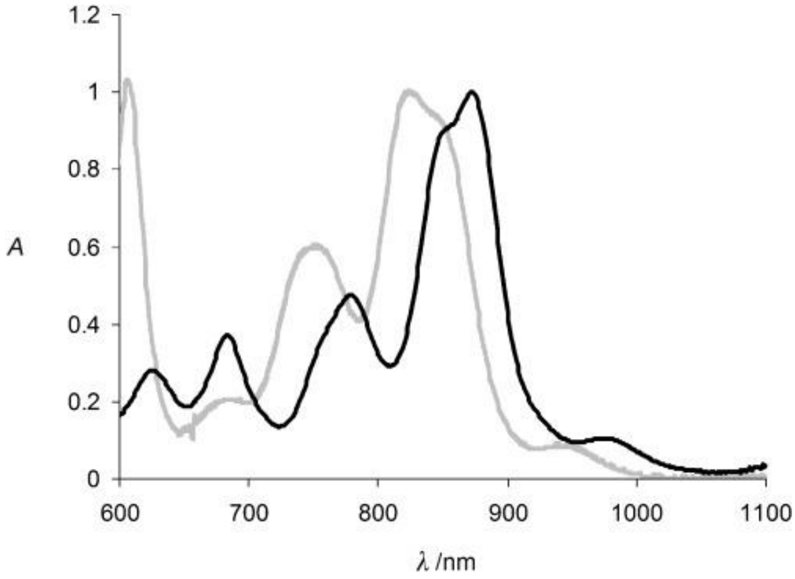

Figure S5. Absorption spectra of two heptacene derivatives reported by Wudl *et al*.[4] Reprinted with permission from John Wiley and Sons (License Number 5975811183536).

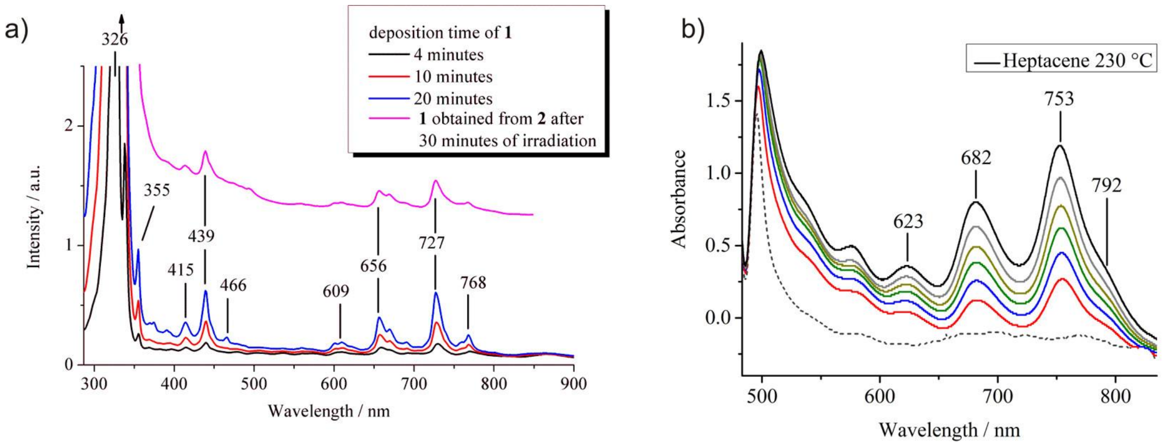

Figure S6. Reprinted with permission from J. Am. Chem. Soc. 2017, 139, 12, 4435–4442.[5] Copyright 2017 American Chemical Society.

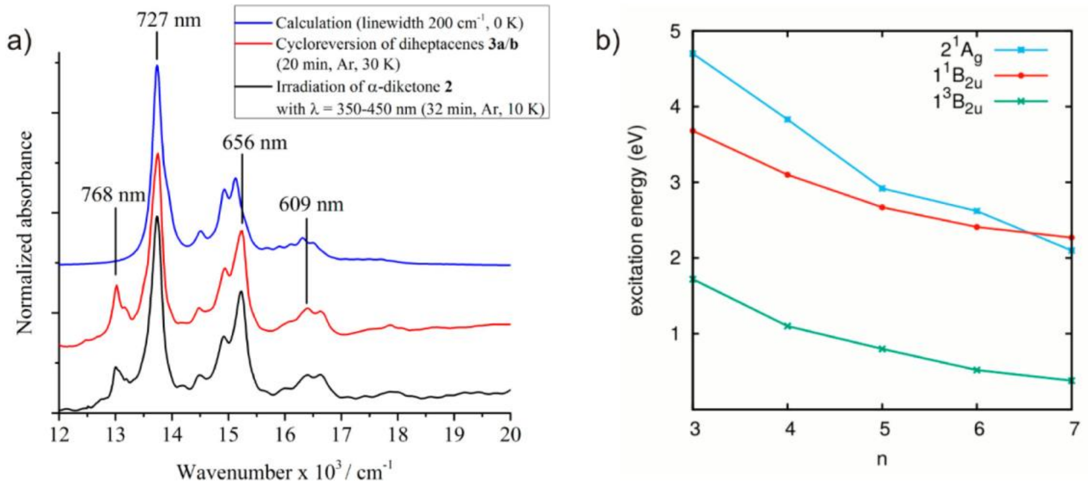

Figure S7. Reprinted with permission from J. Am. Chem. Soc. 2017, 139, 12, 4435–4442.[5] Copyright 2017 American Chemical Society.

Steady state absorption spectroscopy

Absorption spectroscopy was performed on a Lambda 1050 spectrometer from *PerkinElmer* with a *PerkinElmer* 3D WB Det module in the double beam modus. Temperature dependent measurements were performed with an *Oxford Optistat DN2* with a *Mercury iTC* temperature controller. 110-QS cuvettes made of quartz glass from *Hellma* with a light path of 10 mm were used. Typically, all samples were prepared in a glove box under argon atmosphere. Liquid samples were prepared with concentrations of about 10 µmol/L. The solvents applied were at least of HPLC grade and if necessary, they were dried by common lab methods or were taken from a solvent purifications system (MBraun). If not stated otherwise all spectra were recorded at room temperature (20 – 25°C). For the irradiation a medium pressure mercury vapor lamp (USHIO, USH-508S) combined with a dichroic mirror was used.

Solvents in the Vis and NIR:

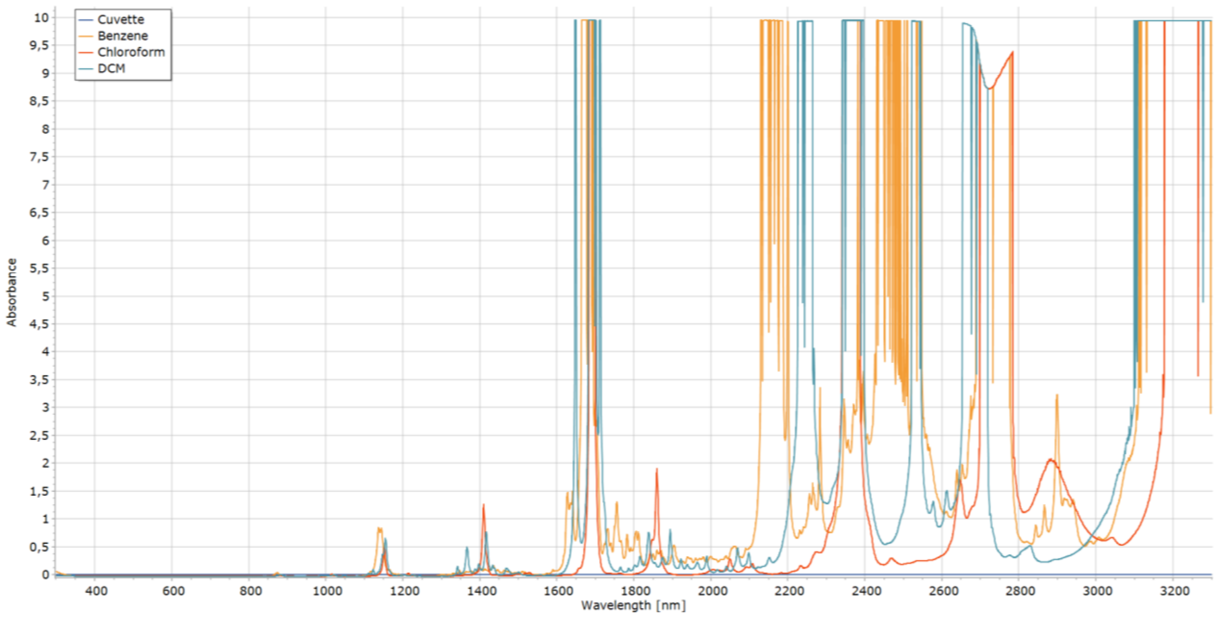

Figure S8. Cuvette and various solvents in the Vis and NIR region.

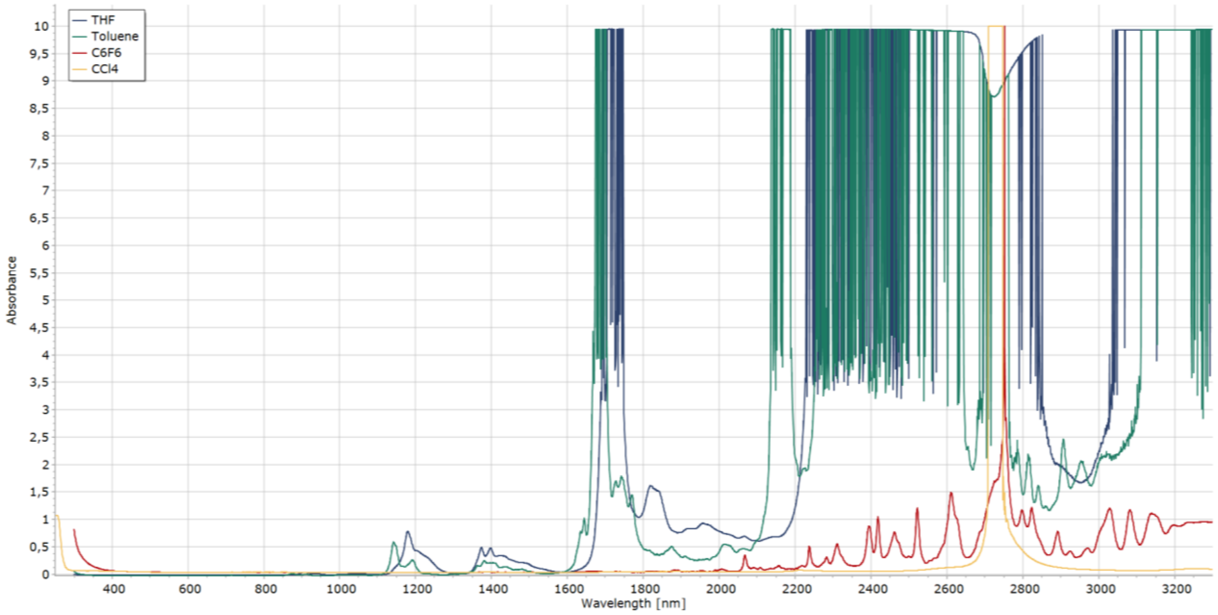

Figure S9. Various solvents in the Vis and NIR region. Solvents without C-H bonds (C_6F_6, CCl_4) are suitable for measurements up to 2000 nm (C_6F_6) and 2600 nm (CCl_4).

Fluorescence spectroscopy

Steady-state spectra were measured on a Horiba Fluorolog-3 spectrofluorimeter equipped with a 450 W xenon lamp for steady-state measurements. Emitted light was detected by a Hamamatsu R13456 PMT (UV/vis/NIR, 200 nm < λ_{em} < 1000 nm) or a Hamamatsu H10330-75 PMT (NIR, 900 nm < λ_{em} < 1700 nm) detector. A double grating monochromator 320DFX (1200 grooves/nm, blazed at 330 nm or 600 grooves/nm, blazed at 750 nm) was used for spectral selection in the excitation path, while in the visible and near infrared emission path the single grating monochromators iHR550 (1200 grooves/mm, blazed at 500 nm; 950 grooves/mm, blazed at 900 nm; 1800 grooves/mm, blazed at 500 nm) and iHR320 (600 grooves/mm, blazed at 1000 nm) were used. To avoid higher order excitation light, long pass filter glass plates (Schott, 3 mm thickness) were used in the emission beam path when needed. Excitation spectra were recorded using two gratings for the excitation wavelength and later merged.

Measurements at 77 K were performed in regular borosilicate glass J. Young NMR tubes (5 mm outer diameter). For cooling and spectrum detection the sample tube within a quartz dewar (Horiba/PTI) filled with liquid nitrogen was placed into the light path. If necessary to reduce artifacts in the spectra from bubbles in the liquid nitrogen at least two consecutive spectra were averaged.

Typically, all samples were prepared in a glove box under argon atmosphere. Liquid samples were prepared with concentrations of about 10 µmol/L or with an absorbance of around 0.1 and if further degassing was necessary, the samples were degassed prior to measurement by at least three pump-freeze-thaw cycles. The solvents applied were at least of HPLC grade and if necessary, they were dried by common lab methods or were taken from a solvent purifications system (MBraun). If not mentioned otherwise a quadratic 1 cm suprasil© quartz cuvette was used for sample solutions measurements in the standard cuvette-holders. If not stated otherwise all spectra were recorded at room temperature (20 – 25°C).

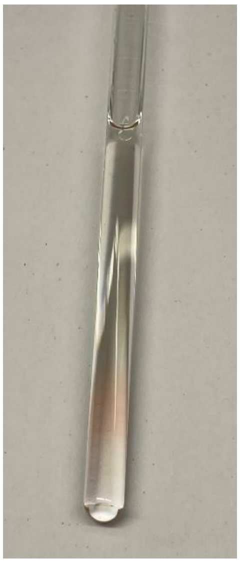

Figure S10. Red coloring of a solution of **TIPS4Hep** in CCl_4 after attempted fluorescence spectroscopy. Only the part where the excitation light beam hit the sample a red coloration is visible.

Transient absorption spectroscopy (TAS)

TAS measurements were performed on a HELIOS Fire fs TA spectrometer and EOS Fire ns spectrometer, both from Ultrafast Systems. The one box femtosecond amplifier Astrella-F-1K Ti:sapphire from Coherent was used to produce 90 fs laser pulses at 800 nm and a repetition rate of 1 kHz. The fundamental beam was split into a probe and a pump beam for the fs TAS experiments, while for the ns experiments, only the pump was used. The wavelength of the pump was mixed to monochromatic 900 nm inside the Apollo-T optical parametric amplifier from Ultrafast Systems using the second harmonic of the idler. For the fs experiments, a white-light continuum probe (WLC) from 340 to 640 nm, 420 to 780 nm and 820 to 1600 nm was generated by focussing the 800 nm probe beam onto a CaF_2 plate, sapphire plate and proprietary plate similar to YAG, respectively. The delay between pump and probe was controlled mechanically by a delay-line from Ultrafast Systems. For the ns experiments, the delay was controlled electronically and a WLC from 380 to 850 nm and 750 to 1600 nm was generated within a photonic crystal fiber.

All samples were prepared under inert conditions in a glovebox. 2 mm quartz glass cuvettes from Hellma were used equipped with a homebuilt screw cap to prevent oxygen infiltration during the measurements. The samples were diluted to approximately 50 µM (0.045 OD at 900 nm) and stirred during the experiments. For the fs TAS experiments, 0.9 µJ per pulse were used while for the ns experiments, the pump power was increased to 8 µJ per pulse to account for the weaker transient signals at longer delay times. We could not observe any non-linear

effects or saturation at these experimental conditions. Steady-state absorbance and emission spectra were taken before and after the experiments to confirm that no degradation happened during the experiments.

The data were exported using the software Surface Xplorer from Ultrafast Systems. Data processing, visualization and fitting were performed using Python 3.11 and libraries such as Matplotlib, NumPy, SciPy and lmfit.[6]

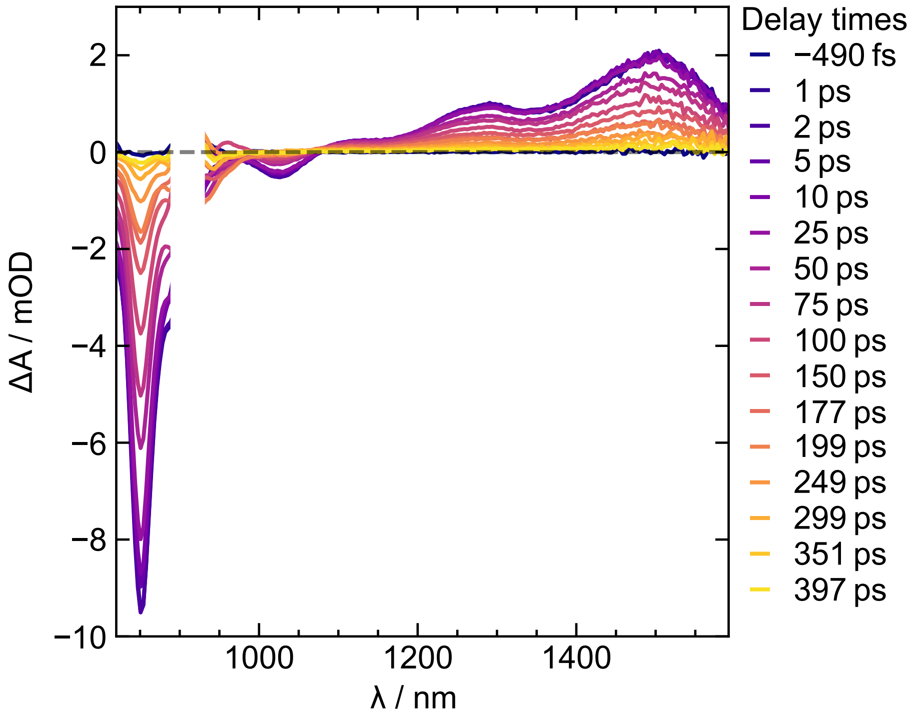

Figure S11. NIR transient absorption spectra of Figure 6a at selected delay times of **TIPS4Hep** in C_6F_6 (λ_{exc} = 900nm, 0.9 µJ / pulse).

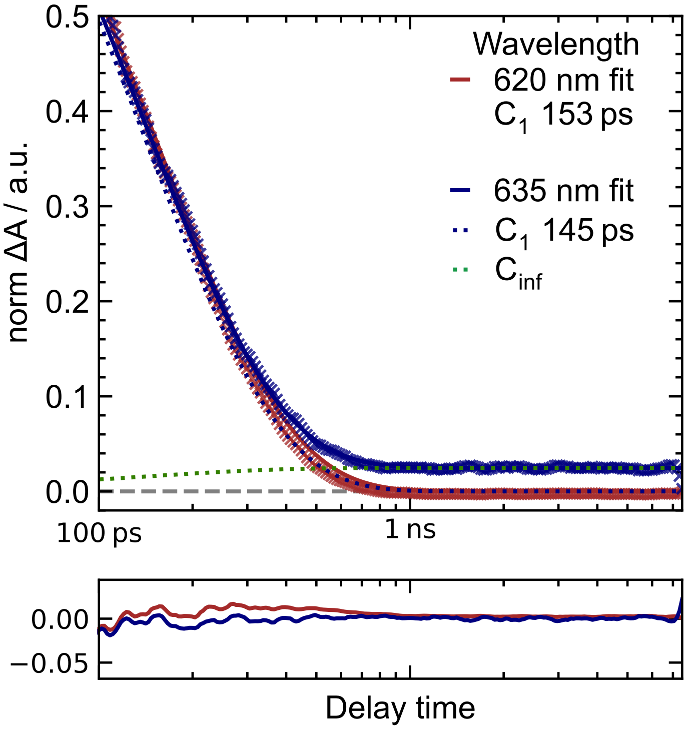

Figure S12. Kinetic traces at 620 nm and 635 nm of Figure 6a in the main manuscript and their corresponding fits (λ_{exc} = 900nm, 0.9 µJ / pulse). and their corresponding fits at selected wavelengths. The residuals of the fits are displayed below the graphs. At 635 nm, a sequential biexponential model is used as explained in the main text.

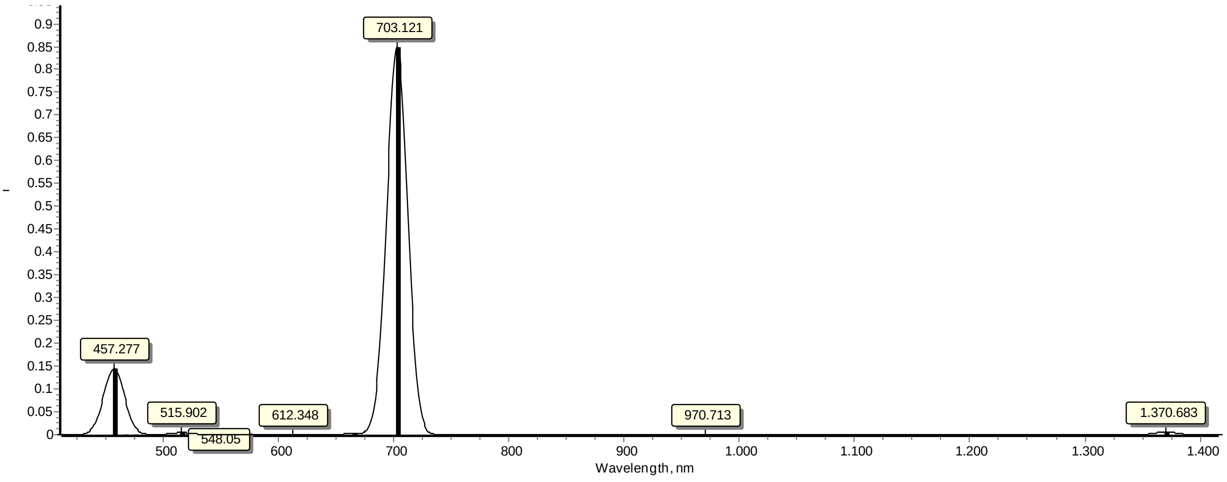

Figure S13. Calculated vertical electronic absorption spectrum of the T_1 state of **TIPS4Hep** (UB3LYP/6-311+G**//UM06-2X/def2-SVP).

Matrix Isolation

Experimental Details

Matrix isolation experiments were performed using standard techniques[7, 8] with a CTI Cryogenics 8200 compressor (Brooks Automation) combined with a CCS-350R vacuum shroud (Janis Research) to achieve temperatures as low as 7 K. The precursor **COHep** was put into a quartz tube that was resistively heated and the sublimation product, heptacene, was deposited along with an excess of argon onto a cold sapphire window (7 K). Argon 6.0 (Westfalen AG, 99.9999%) flow was maintained at 2.0 sccm using a mass flow controller (MKS mass flow PR400B). Quartz external windows were used to measure UV-Vis spectra using a Perkin Elmer Lambda 1050 spectrometer.

Results

Heptacene was produced by heating the precursor at 280°C, leading to decarbonylation and its sublimation. It was thereby deposited on a sapphire window in an excess of argon at 7 K and then characterized by UV-Vis spectroscopy. The UV-Vis spectrum is in good agreement with previous reports on the generation of heptacene via photochemical bisdecarbonylation of a bridged α-diketone precursor.[9]

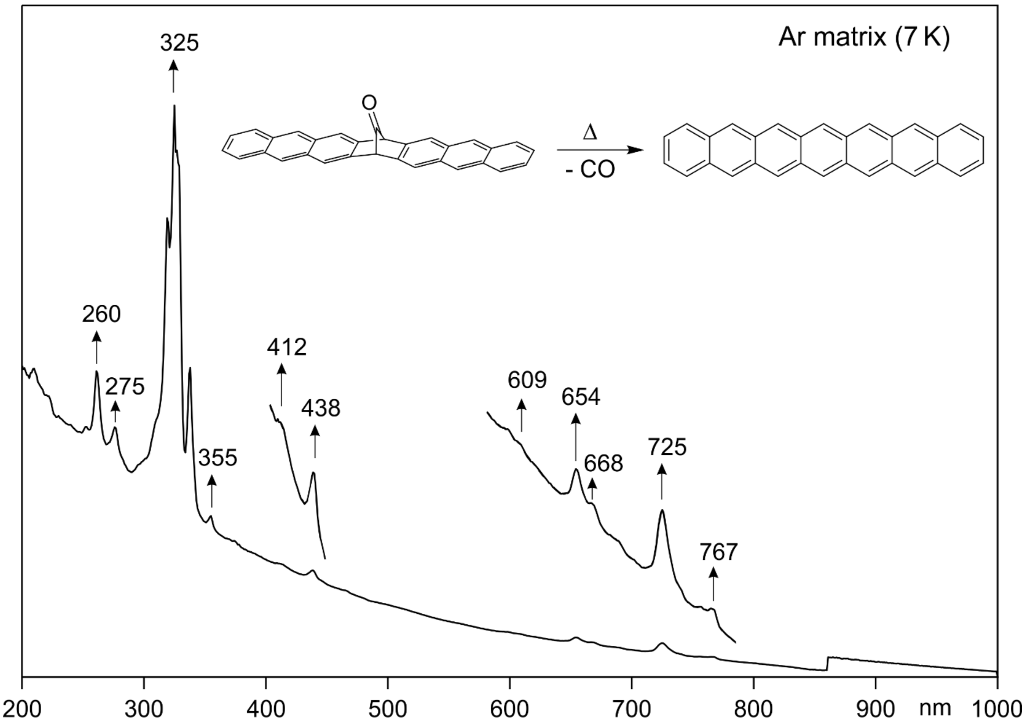

Figure S14. UV-Vis spectrum measured in solid argon at 7 K, showing the formation of heptacene generated from **COHep**.

EPR

EPR experiments have been performed on a *Bruker EMXmicro* with *PremiumX microwave bridge*.

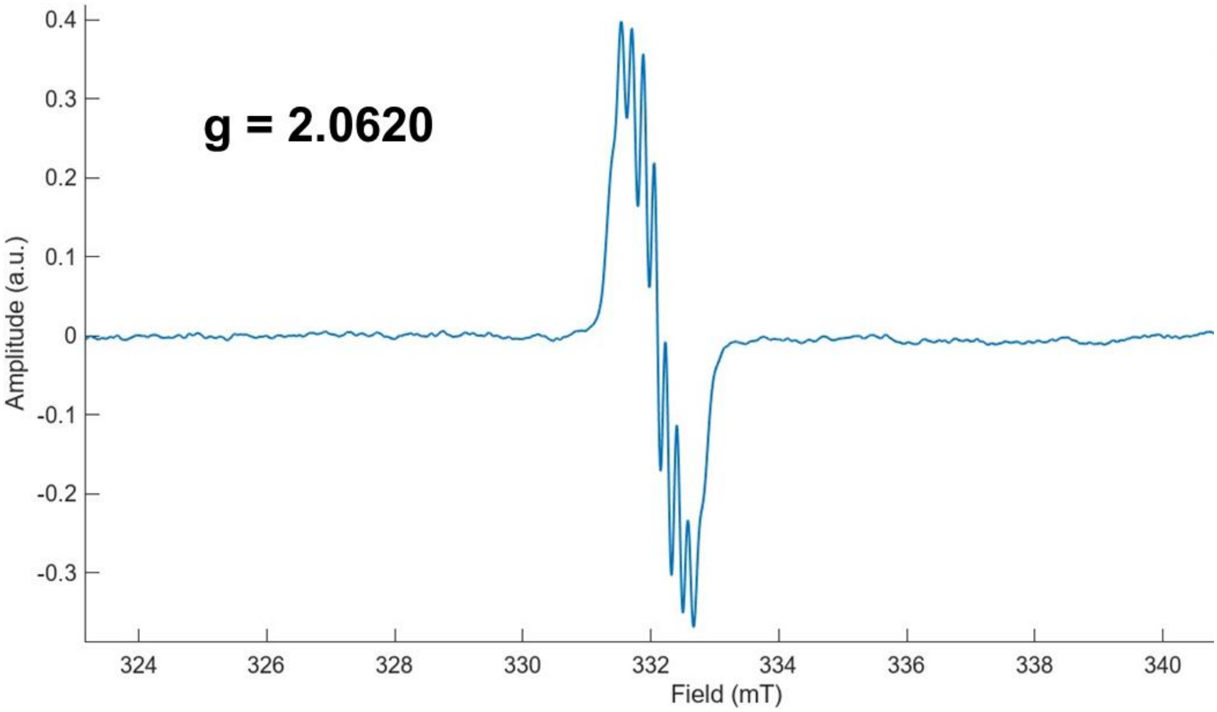

Figure S15. EPR spectrum of radical cation of **TIPS4Hep**, oxidized by $Ag[Al(OC(CF_3)_3)_4$ at rt in C_6F_6.

Computations

TD-UDFT

Electronic absorption spectrum of T_1 of TIPS4Hep

Using the molecular crystal structure as initial guess, the structure of the singlet ground state of **TIPS4Hep** was computed using Gaussian 16[10] and the M06-2X[11] functional using the spin-unrestricted treatment in conjunction with the def2-SVP basis set.[12] The obtained structure was used for the subsequent geometry optimization of the lowest energy triplet state of **TIPS4Hep** at UM06-2X/def2-SVP. Likewise, the geometry of the T_1 state (1^3B_{2u}) of heptacene was computed at the M06-2X/def2-SVP level. Computation of harmonic vibrational frequencies confirmed that the triplet structures correspond to minima. The excitation energies were calculated using the UB3LYP[13, 14] functional in conjunction with the 6-311+G** basis set.[15]

The electronic absorption spectrum of the lowest energy triplet state of heptacene was computed previously using the PPP-MRSDCI approximation, and it was found that double excitations are important for some of the excited states.[Chakraborty JCP2014, https://doi.org/10.1063/1.4897955] As double excitations are challenging to deal with in TD-DFT, we computed parent heptacene for comparison. At UB3LYP/6-311+G**//M06-2X/def2-SVP, the strong feature (oscillator strength f = 1.14) is at 601 nm (2.06 eV) that agrees well with the previous PPP-MRSDCI and experimental results (both 580 nm)[Chakraborty JCP2014, https://doi.org/10.1063/1.4897955; Mondal 2008, http://pubs3.acs.org/acs/journals/doilookup?in_doi=10.1021/jp076738l] despite some large de-excitation value associated with this excited state. The corresponding excited triplet state of **TIPS4Hep** is at 703 nm (1.76 eV, f = 0.85) and has large de-excitation values. We think that the trend of bathochromic shift of the intense band by around 0.2 eV observed in our experiment compared to that of Mondal et al.[Mondal2008] due to the TIPS groups is nonetheless supported by the TD-DFT calculations.

Lowest energy triplet state geometry of TIPS4Hep (UM06-2X/def2-SVP)

```
176
SCF Done:  E(UM062X) =  -4032.99201873     A.U. after    7 cycles
 C                 -0.71065000    8.61455500   -0.37890800
 C                 -1.40662800    7.42785700   -0.31512700
 C                 -0.71849700    6.18213200   -0.24823700
 C                  0.71854100    6.18213000   -0.24817300
 C                  1.40668100    7.42785300   -0.31500200
 C                  0.71071300    8.61455300   -0.37884500
 C                 -1.39706100    4.94859800   -0.18263800
 C                  1.39709500    4.94859300   -0.18252100
 C                  0.72305600    3.72751600   -0.12115000
 C                 -0.72303300    3.72751700   -0.12120200
 C                 -1.43630500    2.46830400   -0.05706200
 C                 -0.72963200    1.23949000   -0.03116300
 C                  0.72963500    1.23948700   -0.03114400
 C                  1.43631700    2.46829900   -0.05698200
 H                 -2.48163800    4.94900100   -0.18296900
```

H	-1.24649200	9.55787100	-0.42960500
H	-2.49341100	7.42563200	-0.31510900
H	2.49346500	7.42562500	-0.31488900
H	1.24656200	9.55786700	-0.42949400
H	2.48167300	4.94899200	-0.18276000
C	-0.72963800	-1.23951200	0.03104400
C	-1.43631700	-2.46832300	0.05698200
C	-0.72305200	-3.72753500	0.12122200
C	0.72303800	-3.72754000	0.12117900
C	1.43630500	-2.46833200	0.05691500
C	0.72962900	-1.23951700	0.03103100
C	-1.39708600	-4.94860700	0.18275600
C	1.39707000	-4.94861600	0.18265500
C	0.71850900	-6.18214400	0.24840900
C	-0.71852800	-6.18213900	0.24846400
C	-1.40666500	-7.42785500	0.31546000
H	-2.49344900	-7.42562400	0.31543600
C	-0.71069400	-8.61455000	0.37935000
C	0.71066900	-8.61455500	0.37929500
C	1.40664300	-7.42786400	0.31535100
H	-2.48166300	-4.94900400	0.18308000
H	2.48164700	-4.94902000	0.18289800
H	-1.24654100	-9.55785900	0.43012800
H	1.24651300	-9.55786800	0.43003100
H	2.49342700	-7.42564100	0.31524500
C	1.39896300	-0.00001700	-0.00006400
H	2.48330800	-0.00002000	-0.00006200
C	-1.39896600	-0.00001000	-0.00007000
H	-2.48331200	-0.00000700	-0.00007200
C	-2.85345900	2.50391900	-0.01464700
C	-4.07224400	2.63141700	0.03596600
C	-2.85347000	-2.50393500	0.01452900
C	-4.07225400	-2.63142700	-0.03611000
C	2.85345600	-2.50394900	0.01440000
C	4.07223700	-2.63145900	-0.03626500
C	2.85347000	2.50390500	-0.01449500
C	4.07225200	2.63141100	0.03614700
Si	5.89144100	-2.97636300	-0.08853800
Si	5.89144800	2.97635400	0.08845000
Si	-5.89145800	-2.97630200	-0.08854200
Si	-5.89143900	2.97633200	0.08844900
C	-6.01631400	4.86993100	0.39116900
C	-5.45241900	5.29605000	1.76234100
C	-7.41249900	5.48520800	0.16415400
H	-5.35017700	5.28890700	-0.37905400
H	-4.44503900	4.90394200	1.93590400
H	-5.40109900	6.39047300	1.83613000
H	-6.08998400	4.94949300	2.58376000
H	-7.79736400	5.28665200	-0.84090400
H	-8.14805000	5.10758300	0.88224800
H	-7.37223900	6.57559900	0.28752300
C	-6.66668000	1.87268000	1.45786800
C	-8.05644700	2.34483000	1.93618900
C	-5.74142600	1.63656800	2.66929000
H	-6.80412600	0.89919600	0.96077400
H	-8.76085400	2.50441800	1.11376300
H	-8.50120200	1.60068000	2.60953000
H	-7.98543900	3.28275200	2.49819500
H	-4.76966200	1.23205100	2.37266700
H	-5.55653900	2.56479000	3.22160100
H	-6.20269300	0.92852800	3.37046800
C	-6.54500300	2.46096200	-1.64266400
C	-5.99936800	3.36741600	-2.76458600
C	-8.07352500	2.29766800	-1.76019800
H	-6.10081600	1.46444300	-1.78847500
H	-4.90767300	3.44678600	-2.73154900
H	-6.27221400	2.96975800	-3.75075800
H	-6.41122800	4.38172100	-2.70107200
H	-8.46982500	1.57114000	-1.04328600
H	-8.60340600	3.24360100	-1.60174700
H	-8.34256600	1.94361100	-2.76410000
C	-6.54499900	-2.46069800	1.64251200
C	-5.99938400	-3.36702800	2.76454500
C	-8.07351600	-2.29734500	1.76003400
H	-6.10078300	-1.46417400	1.78819600
H	-4.90769200	-3.44643200	2.73151400
H	-6.27221600	-2.96924000	3.75066800
H	-6.41127300	-4.38132900	2.70115700

H	-8.46980000	-1.57090000	1.04303000
H	-8.60342500	-3.24328400	1.60170900
H	-8.34254100	-1.94315100	2.76389100
C	-6.66667900	-1.87280100	-1.45809300
C	-8.05646300	-2.34497200	-1.93634300
C	-5.74142800	-1.63686800	-2.66955200
H	-6.80409500	-0.89925000	-0.96112000
H	-8.76086800	-2.50443900	-1.11389200
H	-8.50120300	-1.60089600	-2.60977400
H	-7.98548500	-3.28296700	-2.49823300
H	-4.76965100	-1.23233900	-2.37298900
H	-5.55657000	-2.56516600	-3.22174600
H	-6.20268100	-0.92890600	-3.37081700
C	-6.01638400	-4.86993500	-0.39102400
C	-5.45249900	-5.29624100	-1.76214200
C	-7.41258500	-5.48514500	-0.16393100
H	-5.35025800	-5.28883200	0.37925200
H	-4.44510700	-4.90418600	-1.93575000
H	-5.40121200	-6.39067500	-1.83579500
H	-6.09005200	-4.94976700	-2.58360400
H	-7.79744600	-5.28645200	0.84110100
H	-8.14812600	-5.10759100	-0.88207300
H	-7.37235500	-6.57555300	-0.28716200
C	6.01632800	-4.86994000	-0.39139300
C	7.41245200	-5.48528400	-0.16420100
C	5.45262800	-5.29593400	-1.76268500
H	5.35005200	-5.28895100	0.37869100
H	7.79715300	-5.28683200	0.84094000
H	7.37217600	-6.57566200	-0.28767700
H	8.14814000	-5.10761700	-0.88213400
H	4.44528800	-4.90378000	-1.93636800
H	6.09033100	-4.94934200	-2.58398100
H	5.40128000	-6.39035000	-1.83656100
C	6.66689500	-1.87261300	-1.45775300
C	5.74182000	-1.63638500	-2.66928900
C	8.05671800	-2.34477500	-1.93590200
H	6.80430100	-0.89916900	-0.96056900
H	4.77002400	-1.23187000	-2.37277200
H	6.20320300	-0.92830000	-3.37034500
H	5.55699200	-2.56455900	-3.22170000
H	8.76099800	-2.50443400	-1.11338100
H	7.98576700	-3.28266100	-2.49797500
H	8.50159600	-1.60059700	-2.60913000
C	6.54475600	-2.46113400	1.64271200
C	8.07326800	-2.29791700	1.76050800
C	5.99890600	-3.36764400	2.76448400
H	6.10059100	-1.46460500	1.78851800
H	8.46972000	-1.57135900	1.04371100
H	8.34215800	-1.94393900	2.76447800
H	8.60313200	-3.24386400	1.60208200
H	4.90721400	-3.44696800	2.73126800
H	6.41073800	-4.38196000	2.70096000
H	6.27161400	-2.97006900	3.75072700
C	6.54477500	2.46131800	-1.64285200
C	8.07329100	2.29815900	-1.76066700
C	5.99889500	3.36792500	-2.76453200
H	6.10063900	1.46479000	-1.78875700
H	8.46976600	1.57153600	-1.04394700
H	8.34219100	1.94429600	-2.76467400
H	8.60312700	3.24410400	-1.60214000
H	4.90720100	3.44721500	-2.73130400
H	6.41069800	4.38224700	-2.70090700
H	6.27161100	2.97045700	-3.75081500
C	6.66693200	1.87248500	1.45755200
C	5.74186500	1.63610700	2.66906500
C	8.05674300	2.34463300	1.93574800
H	6.80436300	0.89909500	0.96026900
H	4.77007800	1.23159700	2.37250600
H	6.20326600	0.92796100	3.37004700
H	5.55701300	2.56421900	3.22157200
H	8.76101900	2.50439300	1.11324200
H	7.98576800	3.28246100	2.49791500
H	8.50164000	1.60039900	2.60890200
C	6.01628800	4.86990300	0.39150000
C	7.41239400	5.48530800	0.16436200
C	5.45258900	5.29574000	1.76284200
H	5.34999400	5.28897700	-0.37853400
H	7.79709100	5.28697400	-0.84080400

H	7.37209000	6.57567300	0.28795300
H	8.14809800	5.10758600	0.88224800
H	4.44526200	4.90353700	1.93649500
H	6.09031000	4.94908300	2.58409700
H	5.40120800	6.39014700	1.83683000

Lowest energy triplet state geometry of heptacene (UM06-2X/def2-SVP)

6	0.000000000	8.585642000	0.710992000
1	0.000000000	7.398014000	2.495715000
6	0.000000000	7.402529000	1.403796000
6	0.000000000	7.402529000	-1.403796000
6	0.000000000	6.153398000	0.716740000
6	0.000000000	8.585642000	-0.710992000
6	0.000000000	6.153398000	-0.716740000
6	0.000000000	4.921757000	1.402219000
1	0.000000000	4.923291000	-2.494990000
1	0.000000000	7.398014000	-2.495715000
6	0.000000000	3.707009000	0.723013000
1	0.000000000	4.923291000	2.494990000
6	0.000000000	2.452813000	1.408091000
6	0.000000000	3.707009000	-0.723013000
1	0.000000000	2.455487000	-2.500597000
6	0.000000000	4.921757000	-1.402219000
6	0.000000000	1.241288000	0.733345000
1	0.000000000	2.455487000	2.500597000
6	0.000000000	0.000000000	1.411973000
6	0.000000000	1.241288000	-0.733345000
6	0.000000000	0.000000000	-1.411973000
6	0.000000000	2.452813000	-1.408091000
6	0.000000000	-1.241288000	0.733345000
1	0.000000000	0.000000000	2.504172000
6	0.000000000	-1.241288000	-0.733345000
1	0.000000000	0.000000000	-2.504172000
6	0.000000000	-2.452813000	-1.408091000
6	0.000000000	-2.452813000	1.408091000
1	0.000000000	-2.455487000	2.500597000
6	0.000000000	-3.707009000	0.723013000
1	0.000000000	-2.455487000	-2.500597000
6	0.000000000	-3.707009000	-0.723013000
6	0.000000000	-4.921757000	1.402219000
6	0.000000000	-4.921757000	-1.402219000
1	0.000000000	-4.923291000	2.494990000
6	0.000000000	-6.153398000	0.716740000
1	0.000000000	-4.923291000	-2.494990000
6	0.000000000	-6.153398000	-0.716740000
6	0.000000000	-7.402529000	1.403796000
6	0.000000000	-7.402529000	-1.403796000
1	0.000000000	-7.398014000	2.495715000
6	0.000000000	-8.585642000	0.710992000
1	0.000000000	-7.398014000	-2.495715000
6	0.000000000	-8.585642000	-0.710992000
1	0.000000000	-9.534688000	-1.248886000
1	0.000000000	-9.534688000	1.248886000
1	0.000000000	9.534688000	1.248886000
1	0.000000000	9.534688000	-1.248886000

Experimental spectra

NMR

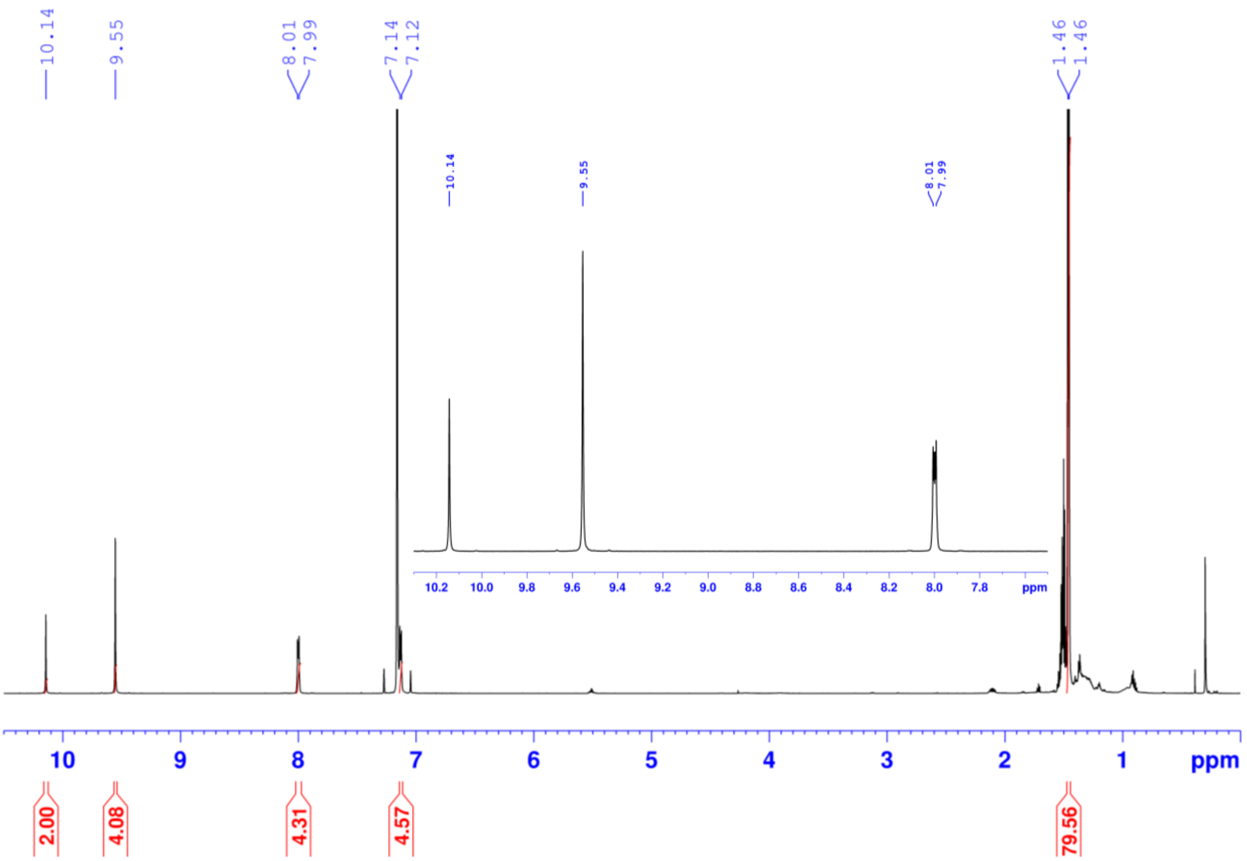

Figure S16. ^{1}H NMR of **TIPS4Hep** (700.29 MHz, C_6D_6).

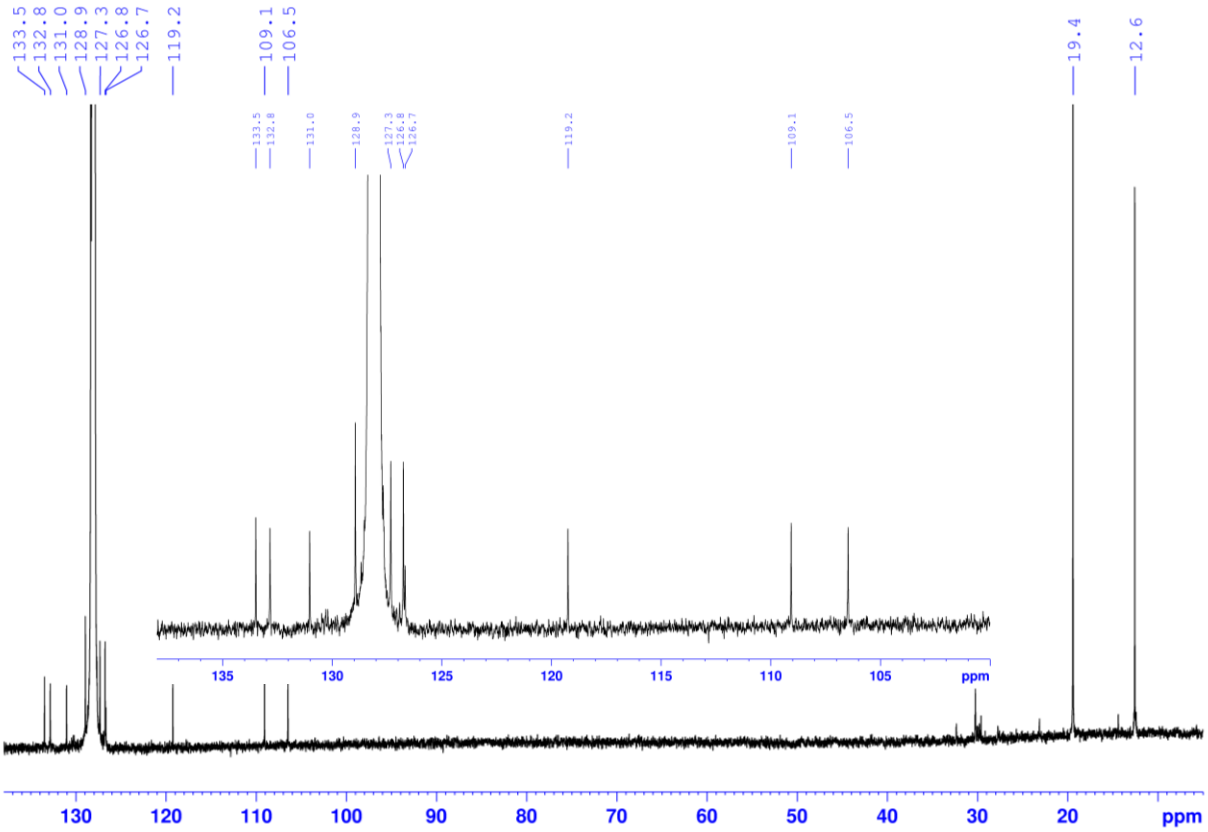

Figure S17. ^{13}C NMR of **TIPS4Hep** (176.09 MHz, C_6D_6).

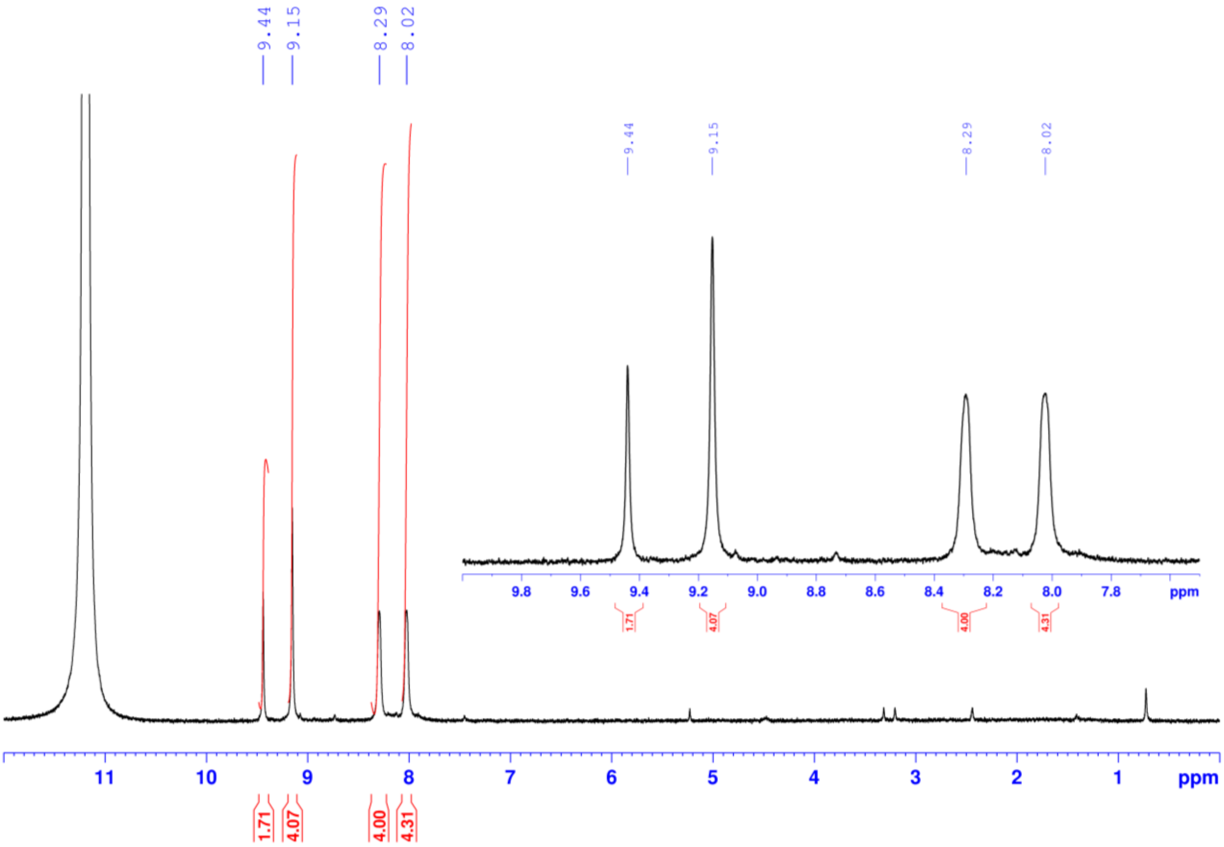

Figure S18. ^{1}H NMR of heptacene-6,8,15,17-diquinone **1** (400.16 MHz, D_2SO_4).

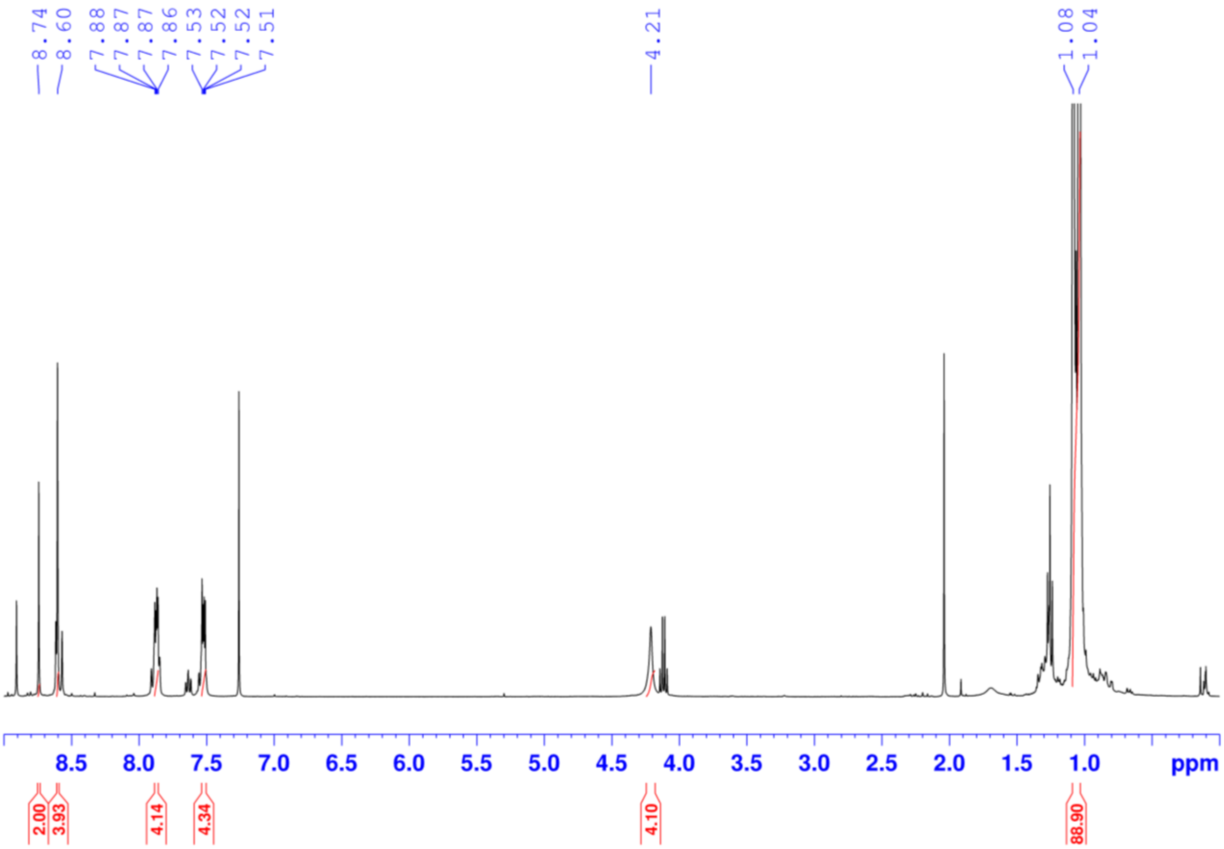

Figure S19. ^{1}H NMR of tetra-6,8,15,17-TIPS-ethinyl heptacene tetrol **2** (400.16 MHz, $CDCl_3$).

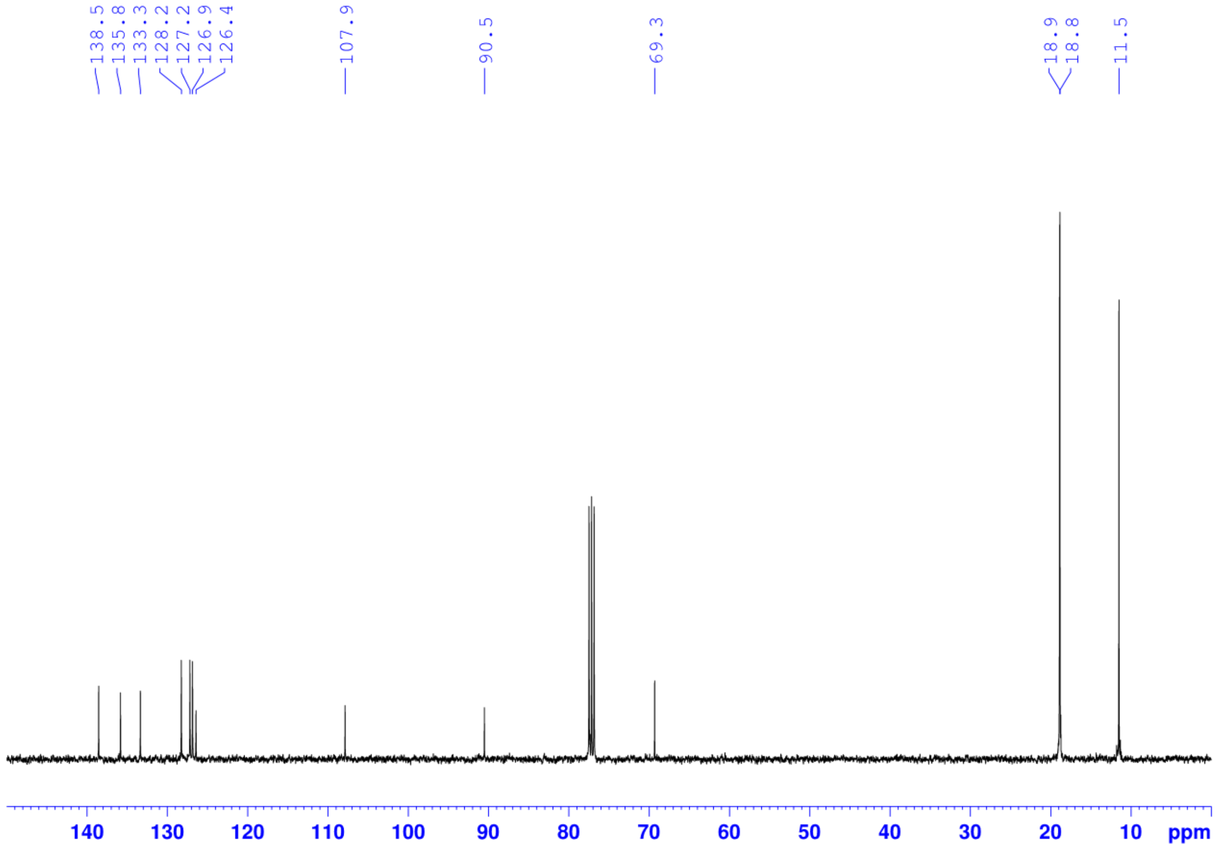

Figure S20. ^{13}C NMR of tetra-6,8,15,17-TIPS-ethinyl heptacene tetrol **2** (400.16 MHz, $CDCl_3$).

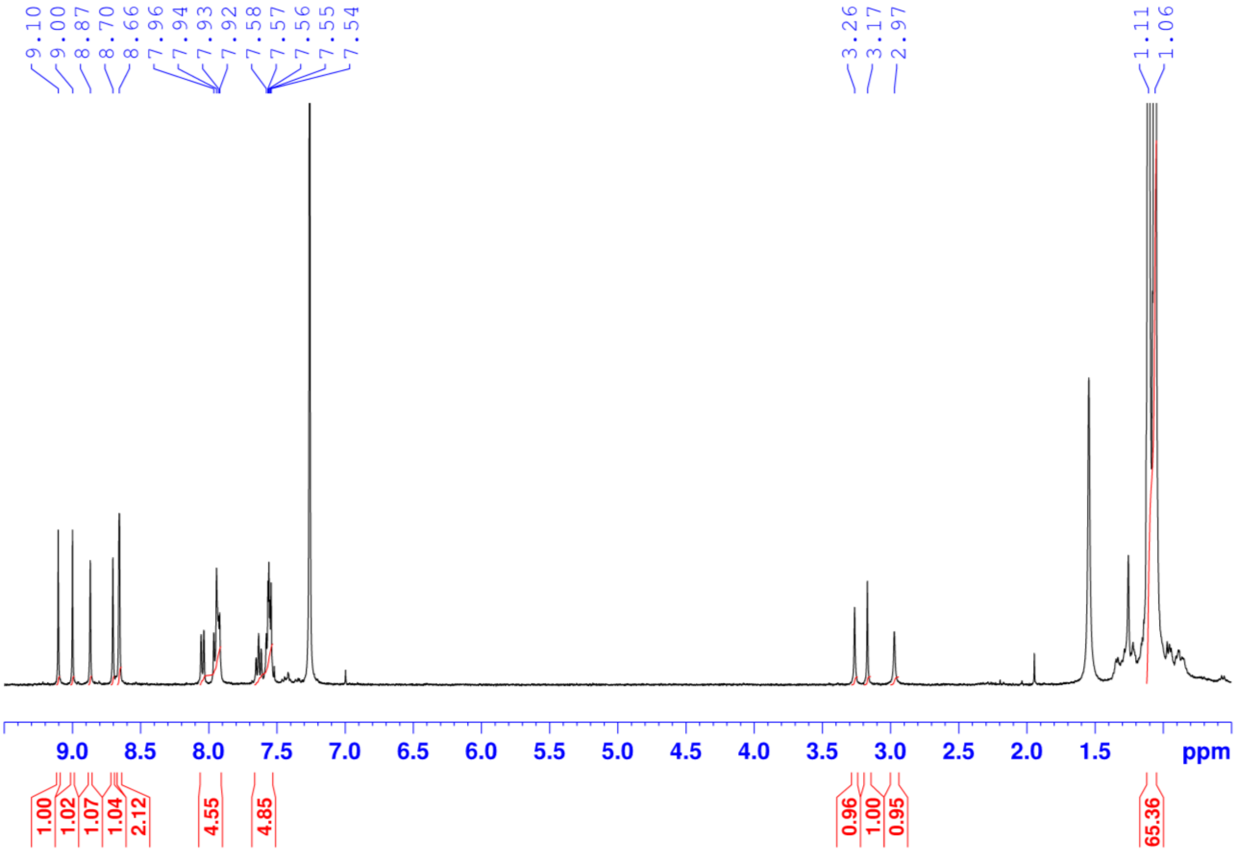

Figure S21. ^{1}H NMR of tri-8,15,17-TIPS-ethinyl heptacene-6-one triol **3** (400.16 MHz, $CDCl_3$).

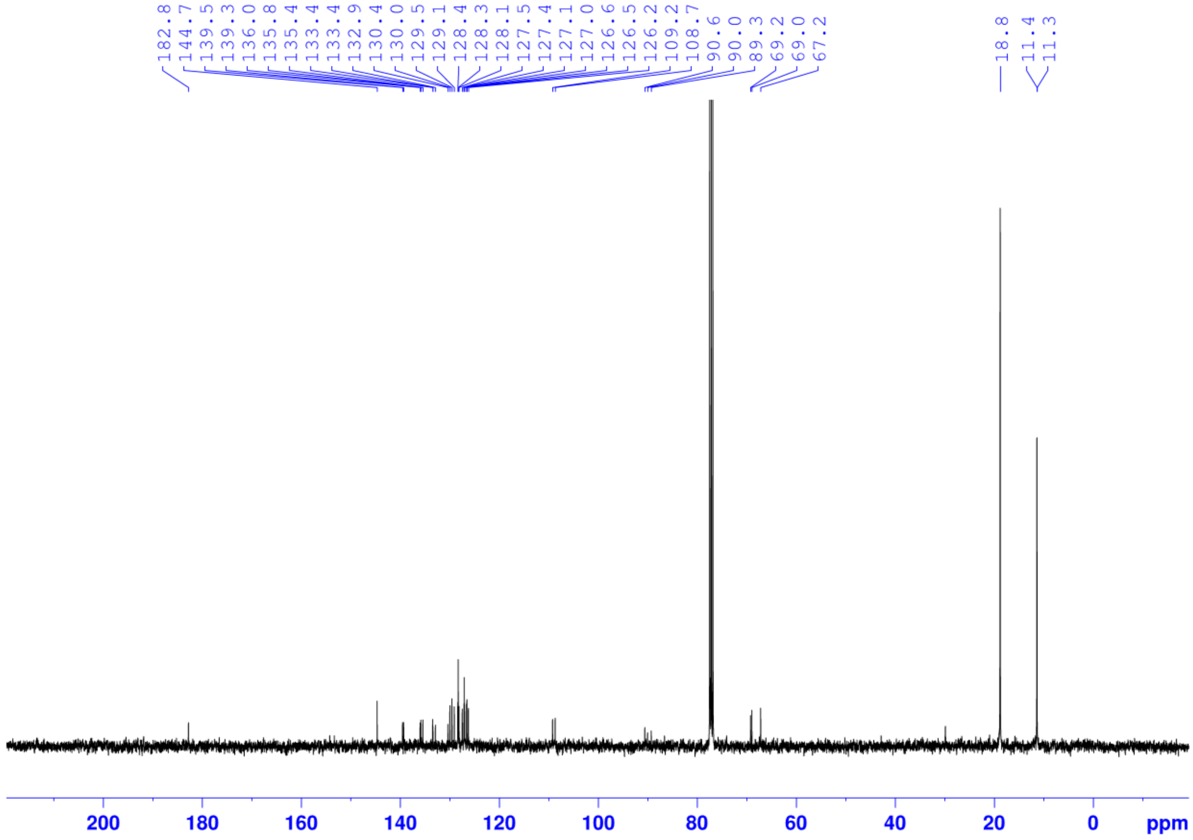

Figure S22. ^{13}C NMR of tri-8,15,17-TIPS-ethinyl heptacene-6-one triol **3** (400.16 MHz, $CDCl_3$).

Mass spectrometry

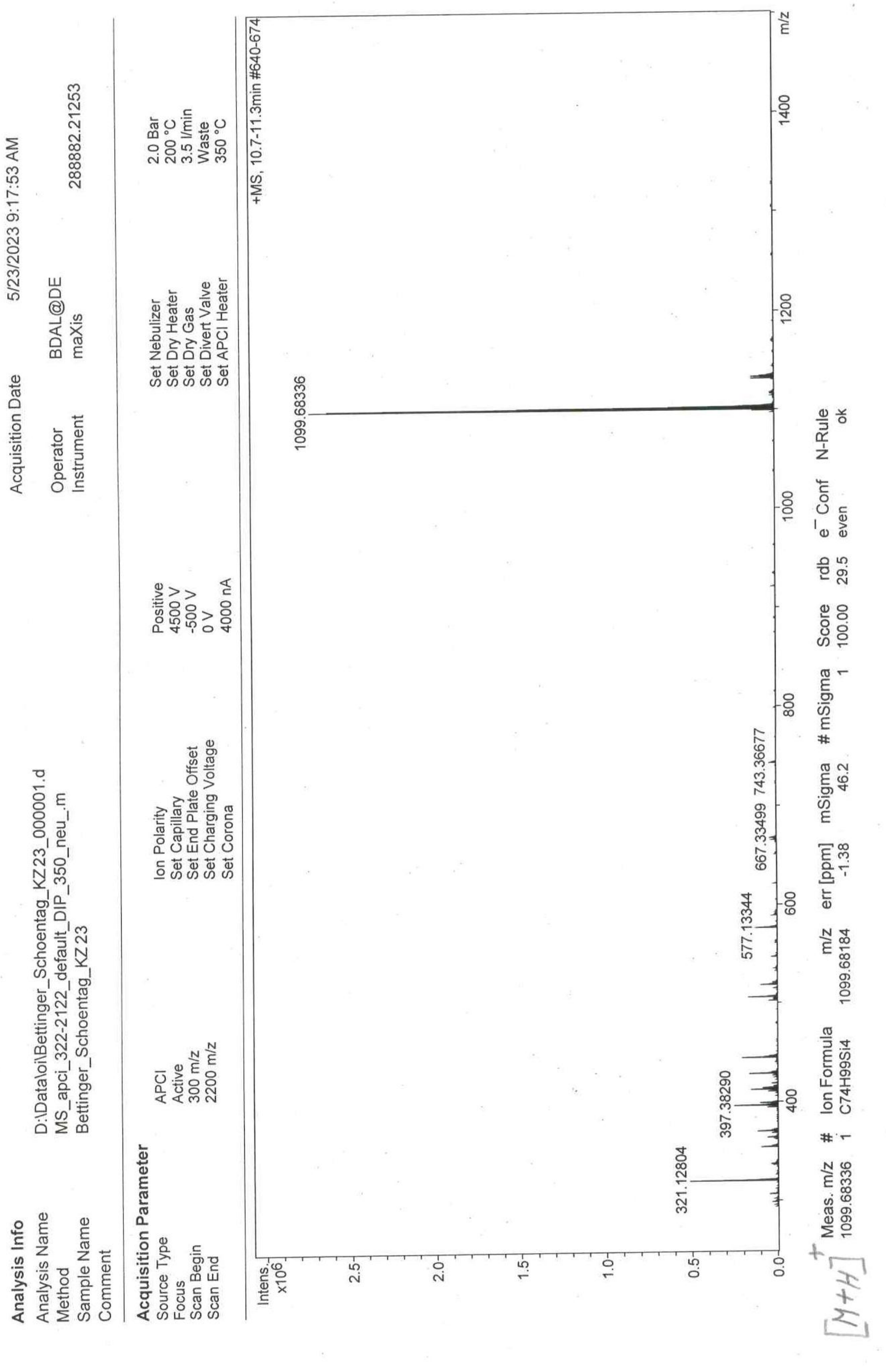

Figure S23. HRMS (APCI) of **TIPS4Hep**.

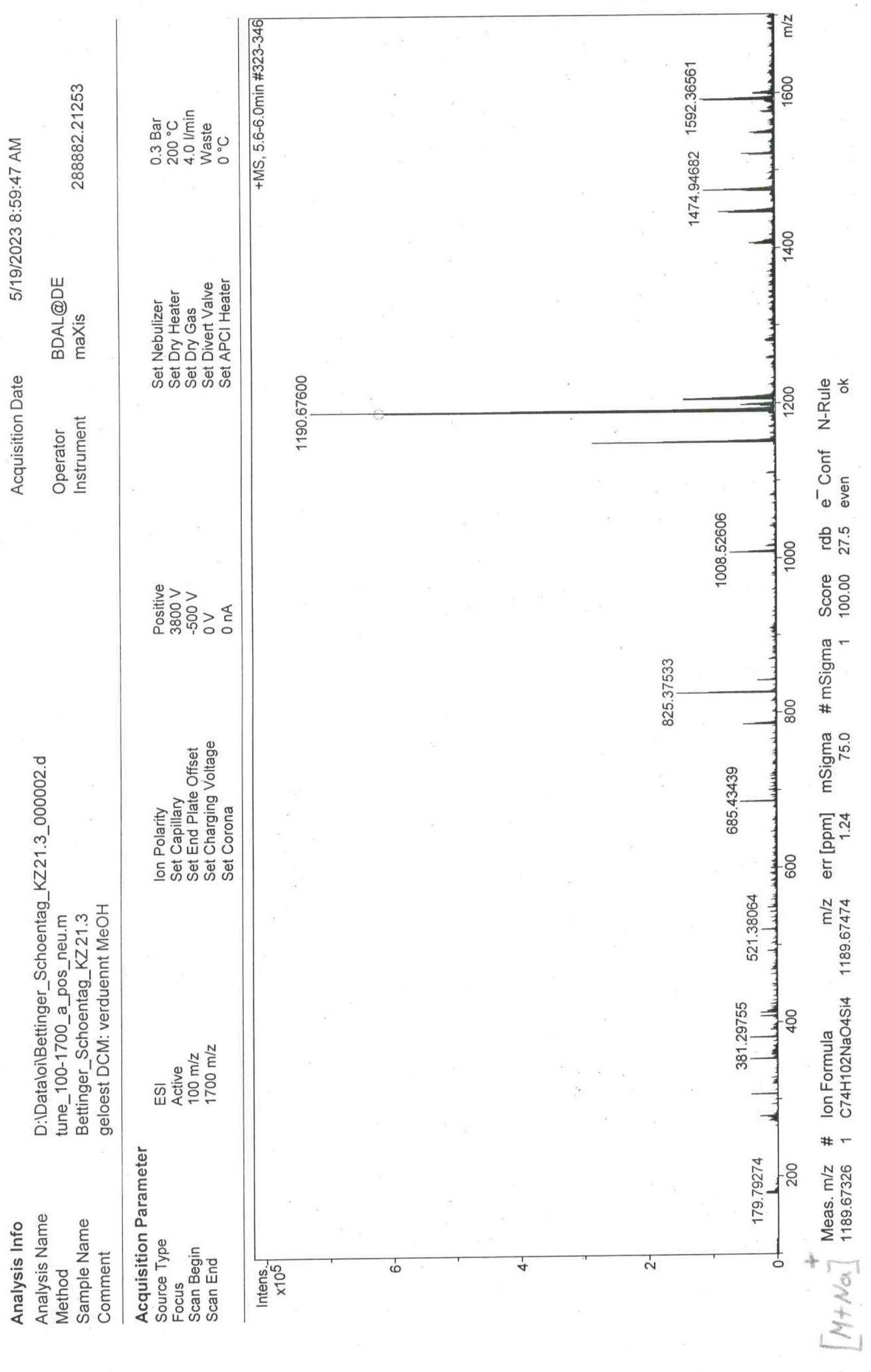

Figure S24. HRMS (ESI) of tetra-6,8,15,17-TIPS-ethinyl heptacene tetrol **2**.

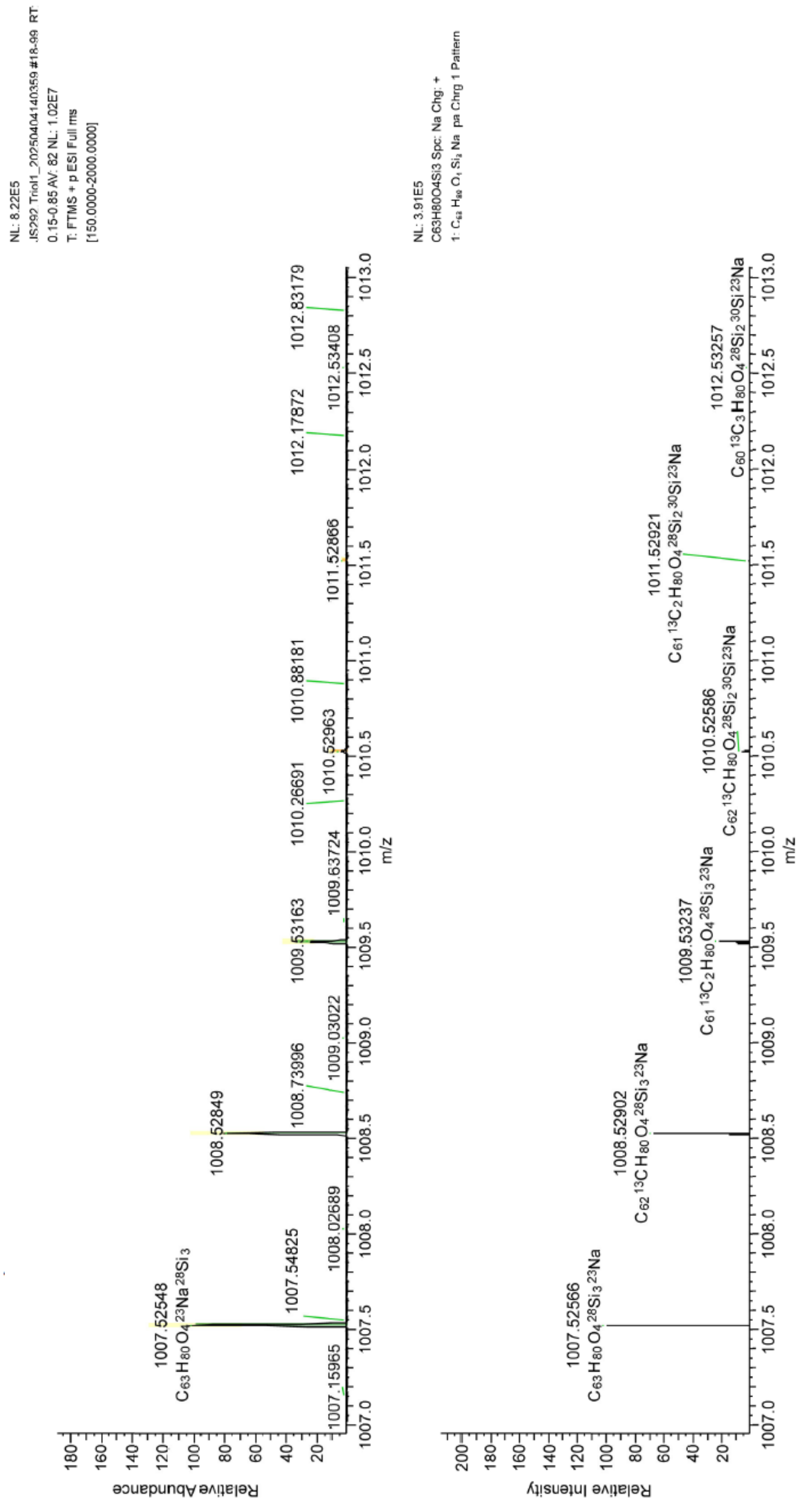

Peak Mass	Display Formula	S Fit	RDB	Delta [ppm]	Theo. mass	Rank	Combined Score	MS Cov. [%]	Pattern Cov. [%]	MSMS Matched Fragments
1007.52548	$C_{63}H_{80}O_4{}^{23}Na^{28}Si_3$	24.3038397403878	26.50	-0.18	1007.52566	1	91.83	95.58	93.55	(Collection)

$[M+Na]^+$ (even)

Figure S25. HRMS (ESI) of tri-6,8,15-TIPS-ethinyl heptacene-6-one triol **3**.

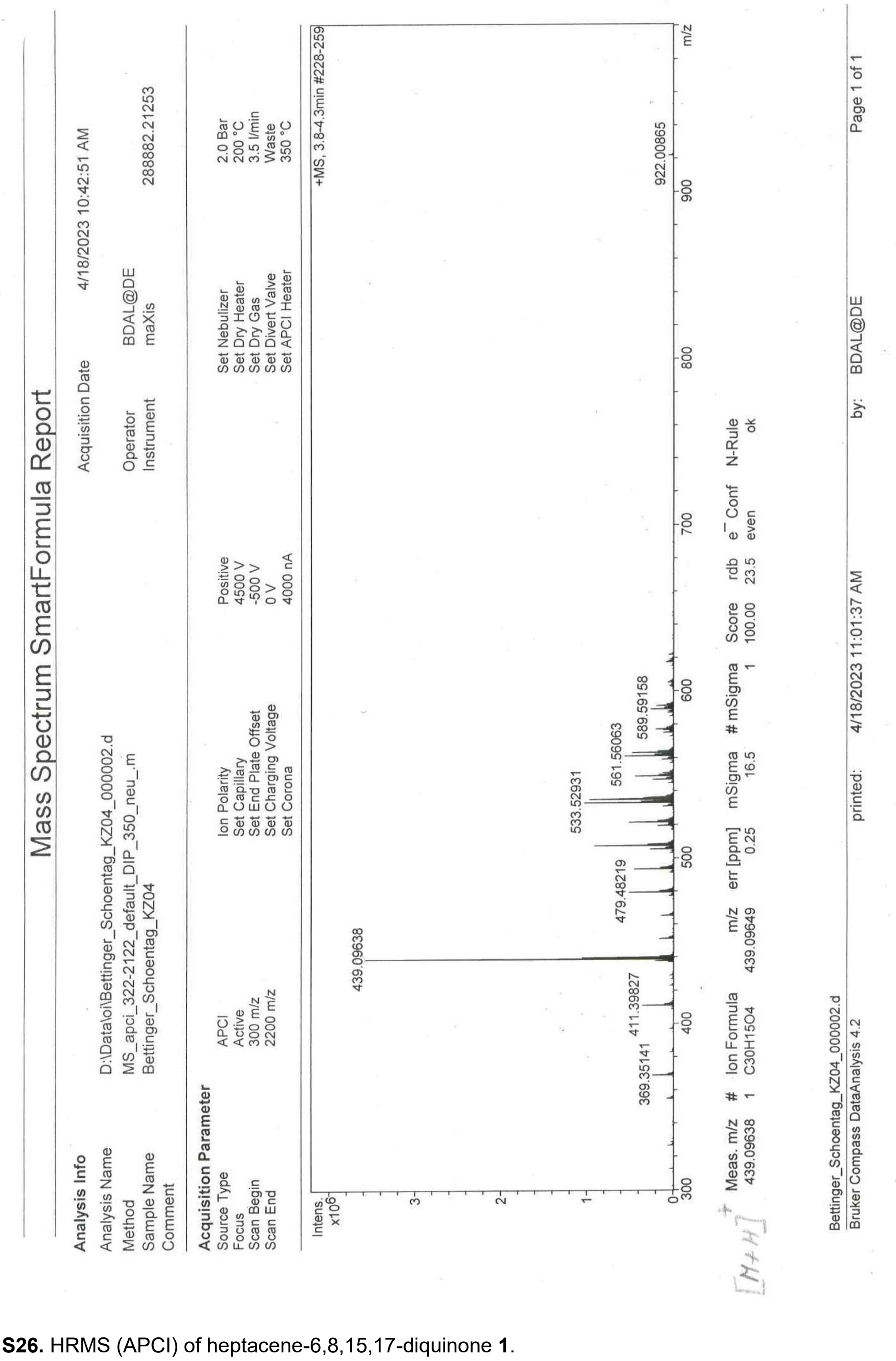

Figure S26. HRMS (APCI) of heptacene-6,8,15,17-diquinone **1**.

References

1. Baxter, I.; Cameron, D. W.; Titman, R. B. Formation of Some Linear Polycyclic Diquinones via a Novel Dimerization. *J. Chem. Soc. C* **1971,** (7), 1253-1256.
2. Jancarik, A.; Levet, G.; Gourdon, A. A Practical General Method for the Preparation of Long Acenes. *Chem. Eur. J.* **2019,** *25* (9), 2366-2374.
3. Payne, M. M.; Parkin, S. R.; Anthony, J. E. Functionalized Higher Acenes: Hexacene and Heptacene. *J. Am. Chem. Soc.* **2005,** *127* (22), 8028–9.
4. Chun, D.; Cheng, Y.; Wudl, F. The Most Stable and Fully Characterized Functionalized Heptacene. *Angew. Chem. Int. Ed. Engl.* **2008,** *47* (44), 8380-5.
5. Einholz, R.; Fang, T.; Berger, R.; Gruninger, P.; Fruh, A.; Chasse, T.; Fink, R. F.; Bettinger, H. F. Heptacene: Characterization in Solution, in the Solid State, and in Films. *J. Am. Chem. Soc.* **2017,** *139* (12), 4435-4442.
6. Newville, M. O., R.; Nelson, A.; Stensitzki, T.; Ingargiola, A.; Allan, D.; Fox, A.; Carter, F.; Michał; Osborn, R.; Pustakhod, D.; Weigand, S.; Ineuhaus; Aristov, A.; Glenn; Mark; mgunyho; Deil, C.; Hansen, A. L. R.; Pasquevich, G.; Foks, L.; Zobrist, N.; Frost, O.; Stuermer; Jaskula, J.-C.; Caldwell, S.; Eendebak, P.; Pompili, M.; Nielsen, J. H.; Persaud, A, *lmfit/lmfit-py: 1.3.2 (1.3.2). Zenodo*, 2024, DOI: https://doi.org/10.5281/zenodo.12785036.
7. Dunkin, I. R. The Matrix-Isolation Technique and Its Application to Organic-Chemistry. *Chem. Soc. Rev.* **1980,** *9* (1), 1-23.
8. Dunkin, I. R., *Matrix-Isolation Techniques: A Practical Approach*. Oxford University Press: **1998**.
9. Mondal, R.; Tönshoff, C.; Khon, D.; Neckers, D. C.; Bettinger, H. F. Synthesis, Stability, and Photochemistry of Pentacene, Hexacene, and Heptacene: A Matrix Isolation Study. *J. Am. Chem. Soc.* **2009,** *131* (40), 14281-14289.
10. Frisch, M. J.; Trucks, G. W.; Schlegel, H. B.; Scuseria, G. E.; Robb, M. A.; Cheeseman, J. R.; Scalmani, G.; Barone, V.; Petersson, G. A.; Nakatsuji, H.; Li, X.; Caricato, M.; Marenich, A. V.; Bloino, J.; Janesko, B. G.; Gomperts, R.; Mennucci, B.; Hratchian, H. P.; Ortiz, J. V.; Izmaylov, A. F.; Sonnenberg, J. L.; Williams; Ding, F.; Lipparini, F.; Egidi, F.; Goings, J.; Peng, B.; Petrone, A.; Henderson, T.; Ranasinghe, D.; Zakrzewski, V. G.; Gao, J.; Rega, N.; Zheng, G.; Liang, W.; Hada, M.; Ehara, M.; Toyota, K.; Fukuda, R.; Hasegawa, J.; Ishida, M.; Nakajima, T.; Honda, Y.; Kitao, O.; Nakai, H.; Vreven, T.; Throssell, K.; Montgomery Jr., J. A.; Peralta, J. E.; Ogliaro, F.; Bearpark, M. J.; Heyd, J. J.; Brothers, E. N.; Kudin, K. N.; Staroverov, V. N.; Keith, T. A.; Kobayashi, R.; Normand, J.; Raghavachari, K.; Rendell, A. P.; Burant, J. C.; Iyengar, S. S.; Tomasi, J.; Cossi, M.; Millam, J. M.; Klene, M.; Adamo, C.; Cammi, R.; Ochterski, J. W.; Martin, R. L.; Morokuma, K.; Farkas, O.; Foresman, J. B.; Fox, D. J., *Gaussian 16 Rev. C.01*, Wallingford, CT, 2016,
11. Zhao, Y.; Truhlar, D. G. The M06 suite of density functionals for main group thermochemistry, thermochemical kinetics, noncovalent interactions, excited states, and transition elements: two new functionals and systematic testing of four M06-class functionals and 12 other functionals. *Theor. Chem. Acc.* **2007,** *120* (1-3), 215-241.
12. Weigend, F.; Ahlrichs, R. Balanced basis sets of split valence, triple zeta valence and quadruple zeta valence quality for H to Rn: Design and assessment of accuracy. *Phys. Chem. Chem. Phys.* **2005,** *7* (18), 3297-305.
13. Becke, A. D. Density-Functional Thermochemistry. III. The Role of Exact Exchange. *J. Chem. Phys.* **1993,** *98* (7), 5648-5652.
14. Lee, C.; Yang, W.; Parr, R. G. Development of the Colle-Salvetti Correlation-Energy Formula into a Functional of the Electron Density. *Phys. Rev. B* **1988,** *37* (2), 785-789.

15. Ditchfield, R.; Hehre, W. J.; Pople, J. A. Self-Consistent Molecular-Orbital Methods. IX. An Extended Gaussian-Type Basis for Molecular-Orbital Studies of Organic Molecules. *J. Chem. Phys.* **1971,** *54* (2), 724-728.
